# The economic burden of malaria: a systematic review

**DOI:** 10.1186/s12936-022-04303-6

**Published:** 2022-10-05

**Authors:** Mônica V. Andrade, Kenya Noronha, Bernardo P. C. Diniz, Gilvan Guedes, Lucas R. Carvalho, Valéria A. Silva, Júlia A. Calazans, André S. Santos, Daniel N. Silva, Marcia C. Castro

**Affiliations:** 1 Centre of Regional Planning and Development (Cedeplar-UFMG), Universidade Federal de Minas Gerais, Belo Horizonte, Minas Gerais Brazil; 2 Centre d’Estudis Demogràfics, CED, Universitat Autònoma B, 08193 Barcelona, Spain; 3 Department of Global Health and Population, Harvard T.H. Chan School of Public Health, Boston, MA 02115 USA

**Keywords:** Malaria, Economic burden, Cost, Cost analysis

## Abstract

**Background:**

Quantifying disease costs is critical for policymakers to set priorities, allocate resources, select control and prevention strategies, and evaluate the cost-effectiveness of interventions. Although malaria carries a very large disease burden, the availability of comprehensive and comparable estimates of malaria costs across endemic countries is scarce.

**Methods:**

A literature review to summarize methodologies utilized to estimate malaria treatment costs was conducted to identify gaps in knowledge.

**Results:**

Only 45 publications met the inclusion criteria. They utilize different methods, include distinct cost components, have varied geographical coverage (a country vs a city), include different periods in the analysis, and focus on specific parasite types or population groups (e.g., pregnant women).

**Conclusions:**

Cost estimates currently available are not comparable, hindering broad statements on the costs of malaria, and constraining advocacy efforts towards investment in malaria control and elimination, particularly with the finance and development sectors of the government.

**Supplementary Information:**

The online version contains supplementary material available at 10.1186/s12936-022-04303-6.

## Background

In 2020, 241 million cases and 627 thousand deaths of malaria were estimated worldwide. Between 2000 and 2015, malaria case incidence reduced by 27%, while the mortality rate reduced by 60%. Since 2015 the decline has slowed (and even reversed in some countries) [1]. Ten countries eliminated malaria between 2000 and 2019, and 21 remained 3 years without an indigenous case. Reducing the malaria burden minimizes out-of-pocket expenses, avoids days lost at school or work due to an infection, and is likely to contribute to economic development. Among countries with intense malaria transmission, the Gross Domestic Product (GDP) grew 1.3% less per person per year, accounting for relevant social and economic factors [2].

Haakenstad et al. [3] estimated that global malaria spending—accounting both for government and out-of-pocket spending—amounted to $4.3 billion (95% UI 4.2–4.4) in 2016, which is an 8.6% (95% UI 8.1–8.9) per year increase over malaria spending in 2000. Estimates were mainly based on national accounts systems from 106 countries and included expenses to prevent and treat malaria. However, macro-analyses such as these, while useful for understanding the landscape of malaria financing, have limited use for strategic planning since they are not able to break down the costs. Quantifying the economic cost of malaria is critical for policymakers to set priorities, allocate resources, select control and prevention strategies, and evaluate the cost-effectiveness of interventions.

Among the principles for a world free of malaria is the stratification by malaria burden, which facilitates optimizing the selection of malaria interventions that are likely to be most effective given the local context. The process of stratification supports decision-making and considers financial resources available for malaria control [4] helping governments to achieve the best outcomes given limited resources. In 2019, the World Health Organization (WHO) Regional Office for Africa proposed a costing tool for countries to plan the budget of their national malaria control programmes [5]. The tool supports budget planning, but it does not assess executed services and does not include indirect costs. Although frameworks for analysis of the economic costs of malaria have been proposed [6–8] comprehensive estimates that break down costs by different stakeholders (health providers, individuals, community), that consider inequities across geographies, and that account for productivity losses and other non-tangible costs are scarce. Only two reviews on the economic burden of malaria are available [7–9]. The first, published in 2003, compiled evidence on direct and indirect costs of malaria for both families and the healthcare system [7]. They estimated monthly per capita expenditures incurred by households for malaria prevention and treatment ranging from, respectively, US$0.05 and US$0.41 in Malawi to US$2.10 and US$3.88 in the urban area of Cameroon. Overall, the average duration of absenteeism due to illness ranged from one to 5 days, reaching 18 days in Ethiopia. Despite detailed estimates of costs, the study was based on critical instead of a systematic review and focused only on African countries. The second, published in 2016, only focused on the economic and financial costs of malaria control, elimination, and eradication, without considering the treatment costs, either direct or indirect [9].

This study aims to conduct a systematic review of the economic burden of malaria. The analysis was conducted to assess whether estimates are available, for different regions where malaria is endemic, considering the perspectives of both individuals and health systems. Also, it aims at appraising the comprehensiveness and comparability of the estimates. Addressing this knowledge gap is important to target public policies that reduce the welfare losses due to malaria. Identifying and mitigating the costs incurred by families is particularly relevant to reducing inequalities.

## Methods

### Study design

A systematic review was conducted to examine the empirical evidence on the economic burden of malaria and its cost components following the principles of the Preferred Reporting Items for Systematic Reviews and Meta-Analysis (PRISMA) statement [10, 11].

### Search strategy and selection criteria

Studies published between 2000 and 2020 (up to May 8) were selected from three scientific articles databases: Medline (via PubMed), Lilacs (via BVS), and Embase. Since the epidemiology of the disease has changed worldwide, the search included only articles published from 2000 onwards to capture the recent trends in the economic burden of malaria. The research question in PECO format (Population, Exposure, Comparator, and Outcomes) and the full search strategy are available in Additional file 1. The terms used were “economics, medical”, “economics, hospital”, “cost and cost analysis”, “cost of illness”, “cost control”, “health care costs”, “health expenditures”, and “malaria” or “paludism”.

References were imported into EndNote X9 [12] and transported into Rayyan for duplicates removal and screening [13]. All references were screened by title and abstract, and those selected had the full texts retrieved and assessed. The exclusion criteria were: (i) cost-effectiveness analyses of treatments, (ii) vaccine efficacy/cost studies, (iii) evaluations of long-term consequences of malaria during childhood, (iv) analyses of specific interventions to control or to eliminate malaria, (v) cost analyses of malaria combined with other infectious diseases that did not allow for the disaggregation of costs by disease, (vi) treatment guidelines, (vii) systematic reviews/literature reviews, (viii) studies without cost components disaggregation or with at most one cost component, and (ix) cost studies about imported malaria. Economic analyses of specific programmes were excluded since they usually report expenditures that are context-related to the intervention. Only full texts were included. No restrictions on language or geographical focus were made.

Seven researchers performed the screening and each paper was independently assessed for inclusion by at least two of them. Any differences were resolved by consensus, following recommendations from the Cochrane Collaboration [14].

### Data extraction and analysis

A qualitative synthesis of the results was performed by systematically organizing the information extracted from the included studies. Data extracted included country and year of study, year of publication, currency, cost components, cost disaggregated by selected attributes (age and severity of the disease), source of research funding, and perspective of the study (healthcare system, household, and societal). A societal perspective is a comprehensive approach that considers healthcare system costs and direct and indirect household costs. The economic costs often fall into two categories. First, direct costs that include medical (treatment and control) and non-medical (transport, lodging, and food) expenses. Second, indirect costs that include absenteeism (short-term absence from work or school due to health problems), presenteeism (reduced performance while working or at school due to health problems), and value of lost time due to morbidity or premature mortality. Economic burden estimates are a broad framework to evaluate the wellbeing impacts as it considers all the economic costs associated with the disease. A broader perspective includes all stakeholders, and the costs are presented as the share of the gross domestic product. Other estimates consider all expenses financed by a specific agent, such as families. In this case, the economic burden is defined as a proportion of the household budget.

One researcher performed data extraction, which was then checked by other two. Information on cost components sought in each study is detailed in Additional file 2. Average values in local currency were extracted and, to facilitate comparison, all cost values were converted into Purchase Power Parity 2020 American dollars (PPP-USD) using the Campbell and Cochrane Economics Methods Group (CCEMG)—Evidence for Policy and Practice Information and Coordinating Centre (EPPI-Centre) cost converter [15]. Descriptive analyses summarized the main characteristics of the selected studies, and all with valid information were included in the qualitative synthesis.

### Quality assessment

There is no standard method to evaluate the quality of cost studies. Drawing from the available literature [16–18] nine items were selected to assess the reporting quality of the selected studies (Additional file 3). For each item, there were four possible response categories: (i) fully meet the item; (ii) partially meet the item; (iii) did not meet the item; (iv) not applicable. One researcher checked the quality assessment for each paper and any uncertainties were decided by consensus.

## Results

### Study selection

**Fig. 1:**
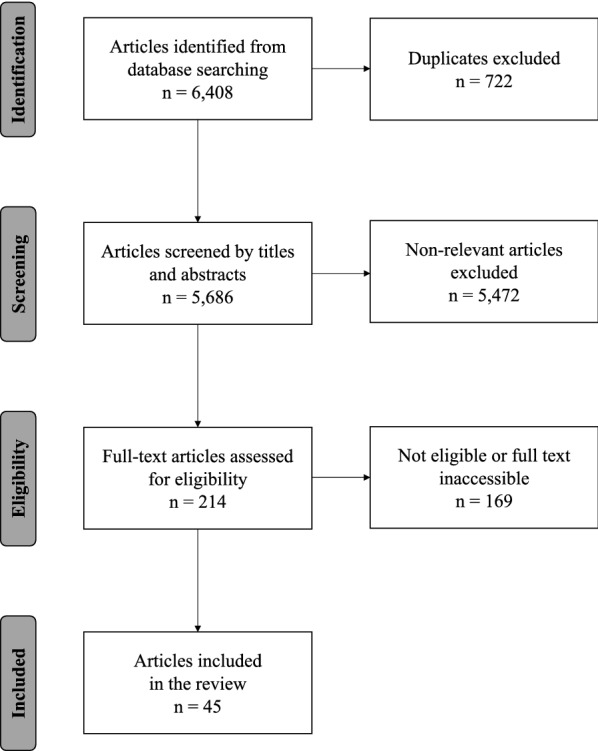
Flow diagram of the systematic review article selection

A total of 6408 articles were initially identified through the database search (Fig. 1). After the removal of 722 duplicates and 5472 references not eligible based on titles and abstracts, 214 studies were suitable for a full review. Following exclusion criteria detailed in Additional file 4, 140 articles were excluded. Despite multiple attempts, it was not possible to obtain the full text of 29 studies (only abstracts were available). The final sample included 45 publications (Additional file 5).

### Characteristics of the studies

**Fig. 2:**
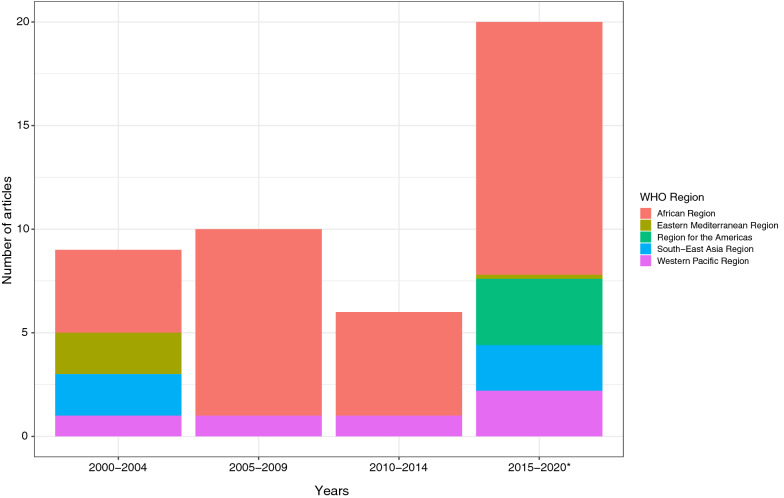
Distribution of studies according to the World Health Organization (WHO) regions from 2000 to 2020. The systematic review covers the period of January 1, 2000, to May 8, 2020. Some studies refer to more than one country

The selected studies analysed 27 countries, almost half from Africa. The most studied countries were Nigeria (n = 7) and Kenya (n = 5). Only three studies involved a cross-country comparison [19–21]. Studies were mostly published between the years 2015 and 2020 (44.4%). From 2005 to 2015 only countries from Africa and the Pacific were investigated, while studies analysing the Americas only appeared after 2015 (Fig. 2).

Most selected studies (76%) received financial support (Additional file 6). The most frequent donors were the World Bank/United Nations Development Programme—UNDP/WHO Special Programme for Training and Research in Tropical Diseases, and the Bill and Melinda Gates Foundation, which funded 20% and 15.5% of the studies, respectively. International health organizations (e.g., WHO) and foreign agencies (e.g., European Union) were important funders for studies in Africa. Only one study was funded by the private sector, specifically the pharmaceutical industry [21].

Two-thirds of the selected papers (67%) carried out the cost analysis using some form of stratification (Additional file 7), such as individual attributes (age and socioeconomic status), access conditions (type of service and distance to the hospital), illness attributes (type of parasite, type of care, and disease severity), endemicity level, seasonality/raining period, and place of residence.

### Qualitative synthesis

There was variation in the cost analysis in terms of the components investigated, source of costs (household or healthcare system), unit of measurement (cost per episode, per household, or per capita), and summary statistics (mean or median). Cost estimates based on household data were the most prevalent (95.5% of the studies), but the number of cost items included varied significantly; 88.9% investigated at least one household direct cost, and 75.6% at least one indirect cost. Medication and treatment/diagnosis were the most reported direct medical cost (Table 1). The majority (n = 41) of the studies reported average cost estimates, and cost per episode (n = 35) was the most common unit of measurement. These differences in estimating and presenting the results limit the comparability of malaria costs across countries.

**Table 1:** Attributes of the studies included (n = 45) in the systematic review

Item/Component	Number of studies	%*
Study perspective		
Healthcare System (only)	2	4.4
Household (only)	33	73.3
Both (Healthcare System and Household)	10	22.2
Societal (include household direct and indirect costs)	7	15.6
Non-Societal (either household direct or indirect costs)	3	6.7
Cost components		
Direct Costs		
Healthcare System		
Prevention	2	4.4
Treatment/Diagnosis	7	15.6
Hospitalization	4	8.9
Medication	6	13.3
At least one component	12	26.7
Household medical direct cost		
Prevention	6	13.3
Treatment/Diagnosis	25	55.6
Hospitalization	11	24.4
Medication	17	37.8
Healers	5	11.1
At least one component	33	73.3
Household non-medical direct cost		
Travelling costs	30	66.7
Food	12	26.7
Lodging	5	11.1
At least one component	31	68.9
At least one direct cost component	40	88.9
Indirect Costs		
Indirect costs—patients		
Travelling (cost of the time)	4	8.9
Medical care	3	6.7
Absenteeism (Days or $)	20	44.4
$ and days	6	13.3
Only days	8	17.8
Only $	6	13.3
At least one component	29	64.4
Indirect costs—caregiver		
Travelling (cost of the time)	2	4.4
Absenteeism (Days or $)	14	31.1
$ and days	8	17.8
Only days	4	8.9
Only $	2	4.4
At least one component	16	35.6
At least one indirect cost component	34	75.6

^*^All percentages were calculated considering the 45 studies

The magnitude of costs depends on several factors such as the healthcare system organization, the level of healthcare coverage, treatment protocols, and private market organization (Table 2). Therefore, estimated costs vary widely, with total and indirect household costs showing the largest variation (e.g., total per capita household costs varied from US$0.48 in Sri Lanka to US$214.68 in India, and total per capita indirect costs was US$0.25 in Kenya and US$182 in India). Patient absenteeism ranged from 1.3 days in Brazil to 11 days in India, while days of caregiver absenteeism varied from 0.2 in Brazil to 9.2 in Malawi.

**Table 2:** Minimum and maximum values of cost components and average absenteeism days per episode

Component	Countries	Costs (PPP values in 2020 US$)		# Studies	References
		Min	Max		
Cost of the healthcare system	Afghanistan, Brazil, Colombia, Ethiopia, Ghana, Indonesia, Kenya, Nigeria, Peru, Philippines, Tanzania, Thailand, and Vietnam	3.91 (Afghanistan)	125.21 (Kenya)	5	[19, 21, 22, 53, 54]
Household costs	Burkina Faso, China, Colombia, Ghana, India, Kenya, Malawi, Mozambique, Myanmar, Nigeria, Papua New Guinea, Peru, Sri Lanka, Tanzania, and Vietnam	0.48 (Sri Lanka)	214.68 (India)	14	[21, 22, 24, 35, 36, 45, 55–61]
Household direct costs	Afghanistan, Brazil, Colombia, Ethiopia, Ghana, India, Indonesia, Kenya, Malawi, Mozambique, Nigeria, Papua New Guinea, Peru, Philippines, Sudan, Thailand, and Vietnam	0.62 (Papua New Guinea, Indonesia)	38.12 (Thailand)	14	[19, 22, 24, 27, 36, 38, 45–47, 51, 54, 59–61]
Household indirect costs	Colombia, Ethiopia, Ghana, India, Kenya, Mozambique, Peru, Sudan, Tanzania, and Vietnam	0.25 (Kenya)	182.37 (India)	10	[21, 22, 24, 37, 45–47, 51, 59, 62]
Days of patient absenteeism	Afghanistan, Angola, Brazil, Colombia, Ethiopia, Ghana, India, Indonesia, Malawi, Mozambique, Myanmar, Peru, Philippines, Thailand, and Vietnam	1.30 (Brazil)	11.00 (India)	11	[19, 20, 24, 36, 45–48, 57, 62, 63]
Days of caregiver absenteeism	Afghanistan, Brazil, Colombia, Ethiopia, India, Indonesia, Malawi, Mozambique, Nigeria, Papua New Guinea, Peru, Philippines, Sudan, Thailand, and Vietnam	0.20 (Brazil)	9.20 (Malawi)	10	[19, 20, 24, 36, 45, 47, 48, 60–62]

In addition to cost components, ten studies estimated the economic burden of malaria. Seven considered a household perspective, two utilized a societal perspective, and one included household and healthcare system costs components but did not consider indirect costs (Table 3). All studies focused on African countries except one that used data from India. Estimates were based on the nominal value of total costs [21, 22], or its share in the gross domestic product (GDP) [23] or the household budget [24–26]. Considering the economic burden of malaria as a percentage of the family budget, results range from 3.12% in India to 8.23% in Nigeria. Catastrophic health expenses due to malaria, measured as the household healthcare expenses exceeding a specified threshold of household income or household capacity to pay, ranged from 17.8% to 22.5% of families in Sudan, Mozambique, and Zimbabwe [20, 27, 28].

**Table 3:** Summary statistics of ten studies that presented economic burden analysis

Country	Perspective	Estimation method	Estimate (% or US$)	Refs.
Malaria expenditure as a percentage of GDP				
Tanzania	Household & Healthcare System	Prevention and treatment of malaria (private, government, and donors) as % of GDP	1.10	[23]
Household economic burden of malaria				
India	Household	% of household income committed to malaria treatment	3.12	[24]
Tanzania	Household	% of household consumption with malaria expenditures	3.30	[26]
Kenya	Household	% of household income committed to malaria treatment	6.50	[51]
Nigeria	Household	% of malaria expenditures in monthly non-food expenditure	8.23	[52]
Percentage of households with catastrophic expenditures due to malaria				
Sudan	Household	Malaria treatment ≥ 15% of monthly income	17.80	[27]
Mozambique	Household	Catastrophic expenditures > 10% of monthly income	17.87	[20]
Mozambique	Household	Catastrophic expenditures > 40% of non-food expenditure	18.50	[20]
Zimbabwe	Household	Catastrophic health expenditures	22.50	[28]
Total expenditures as a proxy for economic burden (US$ of 2020)				
Mozambique	Societal	Unit cost per episode multiplied by the number of cases	345,258	[22]
Ghana	Societal	Total costs of malaria treatment and prevention including costs associated with productivity loss due to death	80,843,886	[21]
Tanzania	Societal	Total costs of malaria treatment and prevention including costs associated with productivity loss due to death	350,786,986	[21]
Kenya	Societal	Total costs of malaria treatment and prevention including costs associated with productivity loss due to death	302,662,132	[21]

Costs were converted into 2020 US dollars using the CCEMG—EPPI-Center Cost Converter [15]

### Quality assessment

Overall, the selected studies met the items that should be presented in economic cost studies (Additional file 8). The description and analysis of the cost components as well as the inclusion of detailed information about the currency and adjustment for inflation were the main limitations encountered.

## Discussion

A systematic review is presented to extract and synthesize evidence on the economic costs of malaria published since 2000. Only 45 publications met the inclusion criteria, out of the 6,408 search results. Most analysed African countries (about 75%), where the highest burden of malaria is concentrated [1]. Indeed, seven of the 11 high-burden countries (which concentrate about 70% of malaria burden) were analyzed: Burkina Faso, the Democratic Republic of the Congo, Ghana, Mozambique, Nigeria, United Republic of Tanzania, and India. While the focus on African countries is not surprising, the scarcity of cost estimates for countries in the Americas and Asia is concerning for at least two reasons. First, as some of those countries approach elimination, cost estimates are critical to inform and sensitize ministers of finance and health to prevent defunding elimination efforts. Second, estimates on costs of malaria as countries approach and achieve elimination will be essential knowledge to high-burden countries in the future. Therefore, cost estimates for countries with diverse transmission intensity remains a gap in the literature.

Global funding has been crucial for the development of studies on the economic costs of malaria with 76% of the selected articles having had received a grant. Three main funders jointly financed 45% of the studies on economic costs of malaria: UNDP/WB/WHO special programme for research and training in tropical diseases, the Bill and Melinda Gates Foundation, and the Wellcome Trust. These results align with previous analyses that show a concentration of the resources in a few funders. Viergever and Hendriks [29] identified the 55 most important public and philanthropic funders. Among the public funders, the US National Institutes of Health ($26.1 billion), the European Commission ($3.7 billion), and the UK Medical Research Council ($1.3 billion) stood out with the highest annual research budgets. The largest philanthropic funders were the Wellcome Trust ($909.1 million), Howard Hughes Medical Institute ($752 million), and Bill and Melinda Gates Foundation ($462.6 million). Official Development Assistance Agencies and Multilateral organizations contributed smaller amounts; the most substantial funding was from the USAID ($186.4 million) and the World Health Organization ($135.0 million). Head et al. showed that between 1993 and 2017, 333 different grants funded malaria-related research investment in sub-Saharan Africa, totaling US$814.4 million [30]. The US National Institutes of Health and the Bill & Melinda Gates Foundation were the main grantors, contributing 60% of the funds [30].

Results shows that there is no systematization of cost components of malaria and no comprehensive and comparable quantification of the economic burden of the disease to society and governments. Comparability of results summarized in this review is difficult because studies vary by the type of cost components included, the estimation method, and the regional level of analysis. Even studies that estimated costs for more than one country have limited comparability [19–21]. Devine et al. [19] is the most comprehensive study, based on a multicentric approach, including nine countries—Afghanistan, Brazil, Colombia, Ethiopia, Indonesia, Philippines, Peru, Thailand, and Vietnam. It investigated direct and indirect household costs for all countries, while costs of treatment and diagnosis from the healthcare system perspective were available only for four countries. The range of estimated values was relatively high, especially for families, with the average total cost varying from US$8.7 in Afghanistan to US$254.7 in Colombia. The main component of the household cost was productivity loss due to illness.

The methodological disparities across selected studies stem from the challenges in investigating healthcare costs. Surveying costs from the provider’s perspective depends on the availability of a systematic and organized information system that stores detailed categories of health spending. Also, some categories of public expenditure may be aggregated for different health conditions or programmes (e.g., surveillance, vector control), making it necessary to implement apportionment strategies to obtain numbers specific for malaria. Similarly, information about the costs associated with the maintenance of the health unit’s physical structure and human resources often must be partitioned among the different diseases based on some criteria [16, 31–34].

Most of the studies included in the review utilized household surveys or interviews with patients/caregivers as the primary sources of information. Therefore, most of the costs were estimated from a household perspective. Still, the comparability of costs across studies is hampered by differences in the cost components included, which varied depending on the specificity of the country/region, the organization of the healthcare system, and the families' vulnerability conditions.

Transportation fees were the most investigated cost component, [19, 22, 24, 35–42] ranging from no cost in the urban area of Benin [43], to US$47.49 for pregnant women who received inpatient care in Manaus, Brazil [42]. High transportation costs in the Brazilian Amazon reflect the long distances that some isolated communities need to travel to receive hospital care [44]. Absenteeism was the second most investigated cost component. In low-income malaria-endemic areas, with precarious labour market conditions, and where family farming is one of the main economic activities, families usually have poor access to social security schemes and incur significant losses in the event of illnesses episodes [45–48]. The review showed that the number of workdays lost per episode of malaria ranged from 1.4 in Brazil [19] to 11 days in high transmission areas of India [24]. Absenteeism days may translate into a high economic burden on families. In Vietnam, workdays lost in the treatment of malaria reached 2% of the total annual household production [45]. Of note is the fact that just one study considered the costs of mortality in the estimates. Potential life-long productivity losses due to premature death were monetized considering the present value of the institutional minimum wage. Considering children aged 0–1 and 1–4 years, the costs of mortality (in thousands) due to malaria was equal to US$ 11.8 and US$ 13.8 in Ghana, US$ 7.6 and US$ 8.9 in Kenya, and US$ 6.9 and US$ 8.1 in Tanzania, respectively [21, 49].

Only eight studies estimated the costs associated with prevention of malaria: six focused on costs incurred by families [24, 26, 37, 42, 48, 50] and two focused on the healthcare system [24, 37]. Since governments are usually in charge of implementing prevention and surveillance actions, costs associated with these strategies should be one of the main components estimated from the healthcare system perspective [3].

Ten studies estimated the economic burden of malaria, but they differ in terms of the perspective and the measurement used to express the economic burden. Three studies considered a broader estimation that included both the household and healthcare system perspectives [21–23]. To assess welfare losses, economic burden should be expressed as the share of the GDP. However, that measure was only calculated by one of those studies that was conducted for Tanzania and considered a comprehensive set of spending from private, government, and international donors [23]. It showed that the burden reached 1.1% of the GDP and 39% of the public spending on health; families bear most of the malaria expenditure (71%), followed by the government (20%). The remaining seven papers conducted a more targeted estimate that computed expenses incurred by households. In this case, the economic burden was expressed as the weight of malaria costs on the household budget or the percentage of families incurring in catastrophic expenditures with results showing that malaria can substantially affect the family’s wellbeing [20, 24, 26–28, 51, 52].

In addition to the limited number of studies that provided estimates on the costs of malaria, the systematic review showed that one of the main limitations was the lack of a standard conceptual framework of the costs to be included and a methodological approach for the calculation. Estimates often vary in several aspects: (i) types of costs considered, (ii) number of years of data used, (iii) choice of cost analysis perspective (healthcare system, household or societal), (iv) geographical coverage, and (v) types of parasites considered. Ideally, the conceptual framework should distinguish different types of costs and stakeholders. Estimates should detail direct and indirect costs and consider differences due to parasite type and spatial geographical heterogeneities in transmission. The cost estimation should also allow its decomposition by different stakeholders (health providers, individual/household, and the community) to target public policies that reduce the welfare losses due to malaria. Identifying the cost components incurred by families is particularly relevant as their economic burden depends on their vulnerability and the organization of the healthcare system. These differences are critical for implementing a proper decision-making process of control strategies and thus must be reflected in cost estimates.

## Conclusion

The limited available estimates are hardly comparable, and there are no comprehensive figures on the cost of malaria from both societal and healthcare system perspectives. This knowledge gap affects proper resource allocation, selection of prevention and control strategies, and evaluation of the cost-effectiveness of interventions. Also, it constrains advocacy efforts towards investment in malaria control and elimination, particularly with the finance and development sectors of the government.

## Supplementary Information

**Additional file 1.** PECO search framework.

**Additional file 2.** Descriptive system of cost components.

**Additional file 3.** Items included in the quality assessment of the included articles.

**Additional file 4.** Studies excluded after full-text review and reasons for exclusion.

**Additional file 5.** List of included references.

**Additional file 6.** Funding sources of studies included in the analysis.

**Additional file 7.** Distribution of selected papers according to the type of stratification.

**Additional file 8.** Quality assessment of the selected papers.

## Data Availability

Not applicable.

## Abbreviations

BVS: Virtual Health Library
CCEMG: Campbell and Cochrane Economics Methods Group
CNPq: Brazilian National Council for Scientific and Technological Development
DECIT: Department of Science and Technology
EPPI-Centre: Evidence for Policy and Practice Information and Coordinating Centre
GDP: Gross Domestic Product
Medline: Medical Literature Analysis and Retrieval System Online
PECO: Population, Exposure, Comparator, and Outcomes
PPP-USD: Purchase Power Parity American dollars
PRISMA: Preferred Reporting Items for Systematic Reviews and Meta-Analysis
SCTIE: Innovation and Health Strategic Products
UNDP/WHO: World Bank/United Nations Development Programme
WHO: World Health Organization
