# Supplementary material for: The economic burden of malaria: a systematic review
Source: Malar J. 2022 Oct 5;21:283. doi: 10.1186/s12936-022-04303-6 (PMC9533489; doi:10.1186/s12936-022-04303-6)
Supplement: Supplementary file 1 — Additional file 1. PECO search framework. [file 12936_2022_4303_MOESM1_ESM.docx]

# Research question

What are the economic costs associated with malaria?

| P – Population | The total population of countries with endemic malaria |
| --- | --- |
| E – Exposure | Malaria |
| C – Comparator | Not Applicable |
| O – Outcomes | Malaria treatment, prevention, surveillance and control costs, indirect costs |

# Database, search strategy and number of references found

| **Database** | **Strategy** | **# ref.** |
| --- | --- | --- |
| Pubmed | ("Economics, Medical"[Mesh] OR "Economics, Hospital"[Mesh] OR "Economics"[Mesh] OR "Costs and Cost Analysis"[Mesh] OR "Cost of Illness"[Mesh] OR "Cost Savings"[Mesh] OR "Cost Sharing"[Mesh] OR "Cost Control"[Mesh] OR "Cost-Benefit Analysis"[Mesh] OR "Cost Allocation"[Mesh] OR "Health Care Costs"[Mesh] OR "Hospital Costs"[Mesh] OR "Health Expenditures"[Mesh] OR disease cost*[tiab] OR cost-analysis[tiab] OR affordab*[tiab]) AND (("Malaria"[Mesh] OR malaria*[ti]) AND (Malaria*[tiab] OR Plasmodium[tiab] OR Remittent Fever[tiab] OR (Fever[tiab] AND Remittent [tiab]) OR Marsh-Fever[tiab] OR Paludism[tiab] OR plasmodi*[tiab] OR Vivax[tiab] OR falciparum[tiab])) | 1,388 |
| Lilacs | (tw:((mh:(Blackwater Fever)) OR (tw:(Blackwater Fever)) OR (tw:(Fiebre Hemoglobinúrica)) OR (tw:(Febre Hemoglobinúrica)) OR (mh:(Malaria)) OR (tw:(Malaria)) OR (tw:(Malária)) OR (mh:(Malaria, Vivax)) OR (tw:(Plasmodium)) OR (mh:(Malaria, Falciparum)) OR (mh:(Malaria, Cerebral)) OR (tw:(Remittent fever)) OR (tw:(Paludism*)) OR (mh:(Malaria, Cerebral)))) AND (tw:((tw:(Economia Médica)) OR (mh:(Economics, Medical)) OR (tw:(Economía Médica)) OR (tw:(Medical Economics)) OR (tw:(Economia da Saúde)) OR (mh:(Health Economics)) OR (tw:(Economía de la Salud)) OR (mh:(Economics)) OR (mh:(Costs and Cost Analysis)) OR (tw:(Costos y Análisis de Costo )) OR (tw:(Custos e Análise de Custo)) OR (tw:(Cost)) OR (tw:(cost anal*)) OR (mh:(Cost of Illness)) OR (tw:(Costo de Enfermedad)) OR (tw:(Efeitos Psicossociais da Doença)) OR (tw:(Burden of Illness)) OR (tw:(Cost of Disease)) OR (tw:(Costs and Cost Analysis)) OR (tw:(Economics, Medical)) OR (tw:(Health Economics)) OR (tw:(Economics)) OR (tw:(Cost of Illness)) OR (mh:(Cost Savings)) OR (tw:(Custo Compartilhado de Seguro)) OR (tw:(Seguro de Costos Compartidos )) OR (tw:(Cost Savings)) OR (mh:(Cost Control)) OR (mh:(Cost Allocation)) OR (mh:(Health Care Costs)) OR (mh:(Hospital Costs)) OR (mh:(Health Expenditures)) OR (tw:(affordab*)))) | 97 |
| Embase | ('malaria'/exp OR 'malaria':ti,ab OR 'malaria infection':ti,ab OR 'malaria transmission':ti,ab OR 'paludism':ti,ab OR 'swamp fever':ti,ab OR 'malaria falciparum'/exp OR 'p. falciparum infection':ti,ab OR 'p. falciparum malaria':ti,ab OR 'plasmodium falciparum infection':ti,ab OR 'plasmodium falciparum malaria':ti,ab OR 'blackwater fever':ti,ab OR 'falciparum infection':ti,ab OR 'falciparum malaria':ti,ab OR 'fever, blackwater':ti,ab OR 'malaria falciparum':ti,ab OR 'malaria tropica':ti,ab OR 'malaria tropicum':ti,ab OR 'malaria, falciparum':ti,ab OR 'pernicious malaria':ti,ab OR 'plasmodium vivax malaria'/exp OR 'p. vivax infection':ti,ab OR 'p. vivax malaria':ti,ab OR 'plasmodium vivax infection':ti,ab OR 'plasmodium vivax malaria':ti,ab OR 'malaria, vivax':ti,ab OR 'vivax infection':ti,ab OR 'vivax malaria':ti,ab OR 'plasmodium'/exp OR 'plasmodium':ti,ab OR 'malaria parasite':ti,ab OR 'plasmodium falciparum'/exp OR 'plasmodium falciparum':ti,ab) AND ('cost'/exp OR 'cost':ti,ab OR 'cost allocation':ti,ab OR 'cost sharing':ti,ab OR 'costs and cost analysis':ti,ab OR 'deductibles and coinsurance':ti,ab OR 'medical savings accounts':ti,ab OR 'cost analysis':ti,ab OR 'cost control'/exp OR 'audit, cost':ti,ab OR 'cost audit':ti,ab OR 'cost containment':ti,ab OR 'cost control':ti,ab OR 'cost savings':ti,ab OR 'cost of illness'/exp OR 'cost of illness':ti,ab OR 'cost of illness analysis':ti,ab OR 'economic aspects of illness':ti,ab) | 5,494 |
| Total |  | 6,979 |
| Total after 1999 |  | 6,408 |
| Duplicates |  | 722 |
| References excluded in phase 2 |  | 169 |
| References included in the final analysis |  | 45 |
