# Supplementary material for: The economic burden of malaria: a systematic review
Source: Malar J. 2022 Oct 5;21:283. doi: 10.1186/s12936-022-04303-6 (PMC9533489; doi:10.1186/s12936-022-04303-6)
Supplement: Supplementary file 2 — Additional file 2. Descriptive system of cost components. [file 12936_2022_4303_MOESM2_ESM.docx]

# Descriptive system of cost components

| **Information to extract** |
| --- |
| Descriptive |
| Authors (year) |
| Paper ID |
| Country |
| Study Year(s) |
| Target population |
| Data source |
| Type of *plasmodium* |
| Currency (year) |
| Exchange rate to the US Dollar |
| Total Cost |
| Costs components |
| Total Direct costs |
| 1 Healthcare system (total) |
| 1.1 Prevention (e.g., mosquito nets, insecticides) |
| 1.2 Diagnosis/Detection (laboratory tests, consultations, and house visits) |
| 1.3 Treatment |
| 1.3.1 Consultations/house visits/ambulatory care |
| 1.3.2 Hospitalizations |
| 1.3.3 Medication/drugs |
| 1.4 Other direct costs (healthcare) |
| 2 Families (total) |
| 2.1 Prevention (e.g., mosquito nets, insecticides) |
| 2.2 Diagnosis (laboratory tests, consultations, house visits) |
| 2.3 Treatment |
| 2.3.1 Consultation/house visits/ambulatory care |
| 2.3.2 Medication/drugs |
| 2.3.3 Hospitalization |
| 2.3.4 Traveling costs (e.g., bus fare) |
| 2.3.5 Food |
| 2.3.6 Lodging |
| 2.3.7 Healers (alternative services) |
| 2.4 Other direct costs (family) |
| Total Indirect Costs (families) |
| 1 Time lost (patients) |
| 1.1 Traveling |
| 1.2 Medical care (consultation, hospitalization, laboratory tests) |
| 1.3a Incapacitation ($)^(1)^ |
| 1.3b Incapacitation (days)^(1)^ |
| 2 Time lost (caregivers) |
| 2.1 Traveling |
| 2.2a Caring for the sick ($)^(2)^ |
| 2.2b Caring for the sick (Days)^(2)^ |
| 3 Other indirect costs |
| 4 Mortality |

(1) Including absenteeism (school/work), performance at work, daily life activities, and permanent incapacity (especially for children)

(2) Including informal caregiver absenteeism and formal caregiver payment (home care)
