# Supplementary material for: The economic burden of malaria: a systematic review
Source: Malar J. 2022 Oct 5;21:283. doi: 10.1186/s12936-022-04303-6 (PMC9533489; doi:10.1186/s12936-022-04303-6)
Supplement: Supplementary file 3 — Additional file 3. Items included in the quality assessment of the included articles. [file 12936_2022_4303_MOESM3_ESM.docx]

**Items included in the quality assessment of the included articles**

- Was the objective of the study clearly stated and properly answered?
- Was the target population of the study clearly described (e.g., gender, age group, regional distribution, or socioeconomic status)?
- Was the study perspective stated?
- Was there a description of the method for cost estimation?
- Were the cost components included in the analysis in line with the perspective adopted in the study?
- Were the cost components clearly described and presented in a disaggregated way to allow for transparency and reproducibility?
- Was there information on the currency and the period in which the costs were collected clearly stated?
- If the costs were collected over different periods, was there an adjustment for inflation? (Not applicable for single-year cross-sectional studies)
- Were the cost components results presented in a disaggregated way?
