# Supplementary material for: The economic burden of malaria: a systematic review
Source: Malar J. 2022 Oct 5;21:283. doi: 10.1186/s12936-022-04303-6 (PMC9533489; doi:10.1186/s12936-022-04303-6)
Supplement: Supplementary file 4 — Additional file 4. Studies excluded after full-text review and reasons for exclusion. [file 12936_2022_4303_MOESM4_ESM.docx]

# Studies excluded after full-text review and reasons for exclusion

| **Reference #** | **Country or region** | **Reason** |
| --- | --- | --- |
| [1] | Brazil | Anthropological study |
| [2] | Sub-Saharan Africa | Cost-effectiveness analysis |
| [3] | 180 countries | Analysis of economic growth |
| [4] | Uganda | Analysis of a specific program, drug, intervention: An economic evaluation of the poor quality of antimalarial |
| [5] | Malawi | Analysis of a specific program, drug, intervention: An economic evaluation of a specific intervention in primary care |
| [6] | Thailand | Cost-effectiveness analysis |
| [7] | Africa | None or at most one cost component |
| [8] | The Democratic Republic of the Congo | Analysis of a specific program, drug, intervention: An economic evaluation of the poor quality of antimalarial |
| [9] | Sub-Saharan Africa | Cost-effectiveness analysis |
| [10] | Sub-Saharan Africa | Analysis of a specific program, drug, intervention: Economic evaluation of training primary healthcare professionals |
| [11] | Uganda | Combined analysis of various diseases |
| [12] | Endemic areas | None or at most one cost component |
| [13] | Kenya | None or at most one cost component |
| [14] | Africa | Literature or Systematic Review |
| [15] | Senegal | Analysis of a specific program, drug, intervention: Economic evaluation of an intervention about malaria drug administration |
| [16] | Pakistan | Cost-effectiveness analysis |
| [17] | Ghana | Cost-effectiveness analysis |
| [18] | Sri Lanka | Analysis of a specific program, drug, intervention: Economic evaluation of an intervention to prevent Malaria |
| [19] | Brazil | Analysis of a specific program, drug, intervention: Economic evaluation about the introduction of a diagnostic test for Malaria |
| [20] | Mauritius, São Tomé and Principe, Swaziland, Tanzania, Mexico, China, India, Indonesia, Iran, Jordan, Lebanon, Philippines, Solomon Islands, Sri Lanka, Syria, Taiwan, Thailand, Vanuatu, Brazil, Colombia | Literature or Systematic Review |
| [21] | Ghana | None or at most one cost component/ Economic evaluation for private sector investment in malaria control |
| [22] | Bhutan | None or at most one cost component |
| [23] | Uganda | None or at most one cost component: Costs of febrile children episode/ it is not specific for malaria |
| [24] | Kenya | None or at most one cost component |
| [25] | Zimbabwe | None or at most one cost component |
| [26] | Brazil | Cost-effectiveness analysis |
| [27] | Myanmar | Analysis of a specific program, drug, intervention: Economic evaluation of the malaria Community Health Worker implementation |
| [28] | Uganda | Willingness to pay analysis |
| [29] | Low- and middle-income countries | None or at most one cost component |
| [30] | Nigeria | None or at most one cost component |
| [31] | 34 countries | Analysis of a specific program, drug, intervention: Analysis of an external donor funding resources allocated for malaria elimination |
| [32] | Tanzania | Cost-effectiveness analysis |
| [33] | Namibia | None or at most one cost component |
| [34] | Burkina Faso | None or at most one cost component |
| [35] | Myanmar | Analysis of a specific program, drug, intervention: Cost analysis of hospitalizations distinguishing by two drug treatments |
| [36] | Bangladesh | Analysis of a specific program, drug, intervention: Economic evaluation of a malaria control program |
| [37] | Zambia | None or at most one cost component |
| [38] | Zambia | Cost-effectiveness analysis |
| [39] | Brazil | Cost-effectiveness analysis |
| [40] | Sub-Saharan Africa | None or at most one cost component |
| [41] | Mali | None or at most one cost component |
| [42] | Africa | None or at most one cost component |
| [43] | Slovak Republic | Cost-effectiveness analysis |
| [44] | Nigeria | None or at most one cost component |
| [45] | Africa | Cost-effectiveness analysis |
| [46] | Endemic areas | Systematic Review of cost-effectiveness studies |
| [47] | Benin, the Democratic Republic of Congo (DRC), Madagascar, Nigeria, Uganda, and Zambia. | Analysis of a specific program, drug, intervention: Analysis of the public and private supply of anti-malarial medicines |
| [48] | Kenya | Analysis of a specific program, drug, intervention: Economic evaluation of school-based intermittent screening and treatment of malaria |
| [49] | Burkina Faso | Cost-effectiveness analysis |
| [50] | Zambia | Cost-effectiveness analysis |
| [51] | Endemic areas | None or at most one cost component |
| [52] | Endemic areas | None or at most one cost component |
| [53] | Mauritius | None or at most one cost component |
| [54] | Sub-Saharan and Southern Africa, South and South-East Asia | Cost-effectiveness analysis |
| [55] | Endemic areas | Analysis of a specific program, drug, intervention |
| [56] | Brazil | Analysis of a specific program, drug, intervention |
| [57] | Zambia | None or at most one cost component |
| [58] | Nigeria | None or at most one cost component |
| [59] | Sub-Saharan Africa | None or at most one cost component |
| [60] | Tanzania | None or at most one cost component |
| [61] | Sudan | Epidemiological study: None or at most one cost component |
| [62] | Africa and a combined America and Central and Southeast Asia regions | Epidemiological study: None or at most one cost component |
| [63] | Slovak Republic | Only imported Malaria |
| [64] | Tanzania | None or at most one cost component |
| [65] | Eritrea | Cost-effectiveness analysis |
| [66] | India | Epidemiological study: None or at most one cost component |
| [67] | Kenya and Uganda | Analysis of a specific program, drug, intervention: intervention to early detection of malaria |
| [68] | Eritrea, Tanzania, Senegal, Malawi, KwaZulu-Natal, Mozambique, Malawi, Togo | Analysis of a specific program, drug, intervention: insecticide-treated net and indoor residual spraying implementation |
| [69] | Endemic areas | None or at most one cost component |
| [70] | India | Epidemiological study: None or at most one cost component |
| [71] | Mozambique | None or at most one cost component |
| [72] | Uganda | Cost-effectiveness analysis |
| [73] | Tanzania | Cost-effectiveness analysis |
| [74] | Global | Epidemiological study: None cost component unspecific for malaria |
| [75] | India | Literature or Systematic Review |
| [76] | Endemic areas | Abstract |
| [77] | 81 malaria-endemic countries | None or at most one cost component |
| [78] | Nigeria | Analysis of a specific program, drug, intervention: Cost of a specific community health workers strategy |
| [79] | Endemic areas | Literature or Systematic Review |
| [80] | Kenya | Combined analysis of various diseases |
| [81] | Togo | Analysis of a specific program, drug, intervention: None or at most one cost component |
| [82] | Nigeria | Willingness to pay analysis |
| [83] | Kenya | Cost-effectiveness analysis |
| [84] | Tanzania | Analysis of a specific program, drug, intervention: Cost of changing national policy guidelines |
| [85] | The Gambia | None or at most one cost component |
| [86] | Sub-Saharan Africa | Cost-effectiveness analysis |
| [87] | Malawi | Analysis of a specific program, drug, intervention: Cost of a nationwide insecticide-treated net program |
| [88] | Europe | Imported Malaria |
| [89] | Brazil | None or at most one cost component |
| [90] | Tanzania | Willingness to pay analysis |
| [91] | Gabon | Abstract |
| [92] | Tanzania | Abstract |
| [93] | Sub-Saharan Africa. | None or at most one cost component |
| [94] | Endemic areas | None or at most one cost component |
| [95] | Sub-Saharan Africa | None or at most one cost component |
| [96] | Endemic areas | Anthropological study |
| [97] | Endemic areas | Review Literature/Systematics |
| [98] | Africa | Cost-effectiveness analysis |
| [99] | Vietnam | None or at most one cost component |
| [100] | India | Epidemiological study |
| [101] | Endemic areas | Abstract |
| [102] | Endemic areas | Literature or Systematic Review |
| [103] | Brazil | Analysis of a specific program, drug, intervention: Cost for community-based malaria management program |
| [104] | Africa | Cost-effectiveness analysis |
| [105] | Endemic areas | Abstract |
| [106] | Africa | Abstract |
| [107] | Global | Epidemiological study: None or at most one cost component |
| [108] | 180 poor countries | Analysis of economic growth |
| [109] | Guyana | Abstract |
| [110] | Africa | Abstract |
| [111] | Africa | Analysis of economic growth |
| [112] | Ethiopia | Abstract |
| [113] | Global | Combined analysis of various diseases |
| [114] | Nigeria | Willingness to pay analysis |
| [115] | Ethiopia | Willingness to pay analysis |
| [116] | Global | Combined analysis for various diseases |
| [117] | Africa | Analysis of a specific program, drug, intervention: Economic evaluation for malaria control |
| [118] | Latin America and the Caribbean | Combined analysis for various diseases |
| [119] | Malawi | Combined analysis for various diseases/ None or at most one cost component |
| [120] | Africa | None or at most one cost component |
| [121] | Kenya | Analysis of a specific program, drug, intervention: cost of prevention campaign of different diseases |
| [122] | Tanzania | Analysis of a specific program, drug, intervention: implementation costs of two different malaria drugs |
| [123] | Tanzania | None or at most one cost component |
| [124] | Ghana | Analysis of a specific program, drug, intervention: Cost of household treatment of presumptive malaria |
| [125] | Burkina Faso | Abstract |
| [126] | Burkina Faso | Abstract |
| [127] | Burkina Faso | Abstract |
| [128] | Ghana | Willingness to pay analysis |
| [129] | Senegal | Analysis of a specific program, drug, intervention: Cost of seasonal malaria chemoprevention in children |
| [130] | Ghana | Abstract |
| [131] | Malawi | Abstract |
| [132] | Global | None or at most one cost component |
| [133] | Nigeria | Abstract |
| [134] | India | None or at most one cost component |
| [135] | Brazil | Abstract |
| [136] | Nigeria | Abstract |
| [137] | Africa | Abstract |
| [138] | Tanzania | Cost-effectiveness analysis |
| [139] | Colombia | Abstract |
| [140] | Nigeria | Abstract |
| [141] | Zambia | Cost-effectiveness analysis |
| [142] | Democratic Republic of Congo | Abstract |
| [143] | Mali | Abstract |
| [144] | Endemic areas | Combined analysis of various diseases/ Literature or Systematic Review |
| [145] | Zambia | Abstract |
| [146] | Brazil | Abstract |
| [147] | Burkina Faso, Nigeria, Uganda | Analysis of a specific program, drug, intervention: Cost of impact of training for community health worker |
| [148] | Kenya | Abstract |
| [149] | Tanzania | Analysis of a specific program, drug, intervention: Cost of seasonal microbial larviciding program |
| [150] | Continents: Americas, Asia, Europe, Middle East and North Africa, Sub-Saharan Africa | Combined analysis of various diseases/ None or at most one cost component |
| [151] | Nigeria | None or at most one cost component |
| [152] | China | Cost for elimination |
| [153] | Countries in the Western Hemisphere | Imported Malaria |
| [154] | India | Abstract |
| [155] | Thailand | Cost-effectiveness analysis |
| [156] | Burkina Faso | Analysis of a specific program, drug, intervention: household costs for children with severe malaria symptoms receiving RA at CHWs *versus* those who did not go to a CHW |
| [157] | Global | Abstract |
| [158] | Afghanistan, Ethiopia, Indonesia, Vietnam | Abstract |
| [159] | Nepal | Combined analysis of various diseases /None or at most one cost component |
| [160] | Nigeria | Abstract |
| [161] | Tanzania | None or at most one cost component |
| [162] | Congo | None or at most one cost component |
| [163] | Myanmar | Abstract |
| [164] | Ghana | Willingness to pay approach |
| [165] | Peru | Literature or Systematic Review |
| [166] | Bolivia | Abstract |
| [167] | Brazil | Abstract |
| [168] | Peru | Cost-effectiveness analysis |
| [169] | 106 countries that were malaria-endemic | None or at most one cost component |

1. Griffing SM, Tauil PL, Udhayakumar V, Silva-Flannery L: **A historical perspective on malaria control in Brazil.** *Mem Inst Oswaldo Cruz* 2015, **110:**701-718.

2. Greenhalgh S, Chandwani V: **Advocating an attack against severe malaria: a cost-effectiveness analysis.** *BMC Public Health* 2020, **20:**17.

3. Sarma N, Patouillard E, Cibulskis RE, Arcand JL: **The Economic Burden of Malaria: Revisiting the Evidence.** *Am J Trop Med Hyg* 2019, **101:**1405-1415.

4. Evans DR, Higgins CR, Laing SK, Awor P, Ozawa S: **Poor-quality antimalarials further health inequities in Uganda.** *Health Policy Plan* 2019, **34:**iii36-iii47.

5. Makaula P, Funsanani M, Mamba KC, Musaya J, Bloch P: **Strengthening primary health care at district-level in Malawi - determining the coverage, costs and benefits of community-directed interventions.** *BMC Health Serv Res* 2019, **19:**509.

6. Sudathip P, Kongkasuriyachai D, Stelmach R, Bisanzio D, Sine J, Sawang S, Kitchakarn S, Sintasath D, Reithinger R: **The Investment Case for Malaria Elimination in Thailand: A Cost-Benefit Analysis.** *Am J Trop Med Hyg* 2019, **100:**1445-1453.

7. Maskin E, Monga C, Thuilliez J, Berthelemy JC: **The economics of malaria control in an age of declining aid.** *Nat Commun* 2019, **10:**2269.

8. Ozawa S, Haynie DG, Bessias S, Laing SK, Ngamasana EL, Yemeke TT, Evans DR: **Modeling the Economic Impact of Substandard and Falsified Antimalarials in the Democratic Republic of the Congo.** *Am J Trop Med Hyg* 2019, **100:**1149-1157.

9. Winskill P, Walker PG, Cibulskis RE, Ghani AC: **Prioritizing the scale-up of interventions for malaria control and elimination.** *Malar J* 2019, **18:**122.

10. Eliades MJ, Alombah F, Wun J, Burnett SM, Clark T, Ntumy R, Chikoko A, Onditi S, Mkomwa Z, Makanka D, Hamilton P: **Perspectives on Implementation Considerations and Costs of Malaria Case Management Supportive Supervision.** *Am J Trop Med Hyg* 2019, **100:**861-867.

11. Soremekun S, Kasteng F, Lingam R, Vassall A, Kertho E, Settumba S, Etou PL, Nanyonjo A, Ten Asbroek G, Kallander K, et al: **Variation in the quality and out-of-pocket cost of treatment for childhood malaria, diarrhoea, and pneumonia: Community and facility based care in rural Uganda.** *PLoS One* 2018, **13:**e0200543.

12. Chitnis N, Schapira A, Schindler C, Penny MA, Smith TA: **Mathematical analysis to prioritise strategies for malaria elimination.** *J Theor Biol* 2018, **455:**118-130.

13. Were V, Buff AM, Desai M, Kariuki S, Samuels A, Ter Kuile FO, Phillips-Howard PA, Patrick Kachur S, Niessen L: **Socioeconomic health inequality in malaria indicators in rural western Kenya: evidence from a household malaria survey on burden and care-seeking behaviour.** *Malar J* 2018, **17:**166.

14. Rogerson SJ, Desai M, Mayor A, Sicuri E, Taylor SM, van Eijk AM: **Burden, pathology, and costs of malaria in pregnancy: new developments for an old problem.** *Lancet Infect Dis* 2018, **18:**e107-e118.

15. Pitt C, Ndiaye M, Conteh L, Sy O, Hadj Ba E, Cisse B, Gomis JF, Gaye O, Ndiaye JL, Milligan PJ: **Large-scale delivery of seasonal malaria chemoprevention to children under 10 in Senegal: an economic analysis.** *Health Policy Plan* 2017, **32:**1256-1266.

16. Howard N, Guinness L, Rowland M, Durrani N, Hansen KS: **Cost-effectiveness of adding indoor residual spraying to case management in Afghan refugee settlements in Northwest Pakistan during a prolonged malaria epidemic.** *PLoS Negl Trop Dis* 2017, **11:**e0005935.

17. Escribano Ferrer B, Hansen KS, Gyapong M, Bruce J, Narh Bana SA, Narh CT, Allotey NK, Glover R, Azantilow NC, Bart-Plange C, et al: **Cost-effectiveness analysis of the national implementation of integrated community case management and community-based health planning and services in Ghana for the treatment of malaria, diarrhoea and pneumonia.** *Malar J* 2017, **16:**277.

18. Shretta R, Baral R, Avancena ALV, Fox K, Dannoruwa AP, Jayanetti R, Jeyakumaran A, Hasantha R, Peris L, Premaratne R: **An Investment Case to Prevent the Reintroduction of Malaria in Sri Lanka.** *Am J Trop Med Hyg* 2017, **96:**602-615.

19. Peixoto HM, Brito MA, Romero GA, Monteiro WM, de Lacerda MV, de Oliveira MR: **Rapid diagnostic test for G6PD deficiency in Plasmodium vivax-infected men: a budget impact analysis based in Brazilian Amazon.** *Trop Med Int Health* 2017, **22:**21-31.

20. Shretta R, Avancena AL, Hatefi A: **The economics of malaria control and elimination: a systematic review.** *Malar J* 2016, **15:**593.

21. Nonvignon J, Aryeetey GC, Malm KL, Agyemang SA, Aubyn VN, Peprah NY, Bart-Plange CN, Aikins M: **Economic burden of malaria on businesses in Ghana: a case for private sector investment in malaria control.** *Malar J* 2016, **15:**454.

22. Wangdi K, Banwell C, Gatton ML, Kelly GC, Namgay R, Clements ACA: **Malaria burden and costs of intensifi ed control in Bhutan, 2006-14: an observational study and situation analysis.** *Lancet Glob Heath* 2016, **4:**e336-e343.

23. Menon MP, Njau JD, McFarland DA, Uganda Malaria Indicator Survey Technical Working G: **Cost and Predictors of Care-Seeking Behaviors Among Caregivers of Febrile Children-Uganda, 2009.** *Am J Trop Med Hyg* 2016, **94:**932-937.

24. Dixit A, Lee MC, Goettsch B, Afrane Y, Githeko AK, Yan G: **Discovering the cost of care: consumer, provider, and retailer surveys shed light on the determinants of malaria health-seeking behaviours.** *Malar J* 2016, **15:**179.

25. Gunda R, Chimbari MJ, Mukaratirwa S: **Assessment of Burden of Malaria in Gwanda District, Zimbabwe, Using the Disability Adjusted Life Years.** *Int J Environ Res Public Health* 2016, **13:**244.

26. Peixoto HM, Brito MA, Romero GA, Monteiro WM, de Lacerda MV, de Oliveira MR: **Cost-effectiveness analysis of rapid diagnostic tests for G6PD deficiency in patients with Plasmodium vivax malaria in the Brazilian Amazon.** *Malar J* 2016, **15:**82.

27. Kyaw SS, Drake T, Thi A, Kyaw MP, Hlaing T, Smithuis FM, White LJ, Lubell Y: **Malaria community health workers in Myanmar: a cost analysis.** *Malar J* 2016, **15:**41.

28. Nanyonjo A, Bagorogoza B, Kasteng F, Ayebale G, Makumbi F, Tomson G, Kallander K, in Ssg: **Estimating the cost of referral and willingness to pay for referral to higher-level health facilities: a case series study from an integrated community case management programme in Uganda.** *BMC Health Serv Res* 2015, **15:**347.

29. Santatiwongchai B, Chantarastapornchit V, Wilkinson T, Thiboonboon K, Rattanavipapong W, Walker DG, Chalkidou K, Teerawattananon Y: **Methodological variation in economic evaluations conducted in low- and middle-income countries: information for reference case development.** *PLoS One* 2015, **10:**e0123853.

30. Etiaba E, Onwujekwe O, Uzochukwu B, Adjagba A: **Investigating payment coping mechanisms used for the treatment of uncomplicated malaria to different socio-economic groups in Nigeria.** *Afr Health Sci* 2015, **15:**42-48.

31. Zelman B, Kiszewski A, Cotter C, Liu J: **Costs of eliminating malaria and the impact of the global fund in 34 countries.** *PLoS One* 2014, **9:**e115714.

32. Maheu-Giroux M, Castro MC: **Cost-effectiveness of larviciding for urban malaria control in Tanzania.** *Malar J* 2014, **13:**477.

33. Smith Gueye C, Gerigk M, Newby G, Lourenco C, Uusiku P, Liu J: **Namibia's path toward malaria elimination: a case study of malaria strategies and costs along the northern border.** *BMC Public Health* 2014, **14:**1190.

34. Bocoum FY, Belemsaga D, Adjagba A, Walker D, Kouanda S, Tinto H: **Malaria prevention measures in Burkina Faso: distribution and households expenditures.** *Int J Equity Health* 2014, **13:**108.

35. Kyaw SS, Drake T, Ruangveerayuth R, Chierakul W, White NJ, Newton PN, Lubell Y: **Cost of treating inpatient falciparum malaria on the Thai-Myanmar border.** *Malar J* 2014, **13:**416.

36. Haque U, Overgaard HJ, Clements AC, Norris DE, Islam N, Karim J, Roy S, Haque W, Kabir M, Smith DL, Glass GE: **Malaria burden and control in Bangladesh and prospects for elimination: an epidemiological and economic assessment.** *Lancet Glob Health* 2014, **2:**e98-105.

37. Comfort AB, van Dijk JH, Mharakurwa S, Stillman K, Gabert R, Korde S, Nachbar N, Derriennic Y, Musau S, Hamazakaza P, et al: **Hospitalizations and costs incurred at the facility level after scale-up of malaria control: pre-post comparisons from two hospitals in Zambia.** *Am J Trop Med Hyg* 2014, **90:**20-32.

38. Sedlmayr R, Fink G, Miller JM, Earle D, Steketee RW: **Health impact and cost-effectiveness of a private sector bed net distribution: experimental evidence from Zambia.** *Malar J* 2013, **12:**102.

39. Oliveira MR, Giozza SP, Peixoto HM, Romero GA: **Cost-effectiveness of diagnostic for malaria in Extra-Amazon Region, Brazil.** *Malar J* 2012, **11:**390.

40. Cohen JM, Woolsey AM, Sabot OJ, Gething PW, Tatem AJ, Moonen B: **Public health. Optimizing investments in malaria treatment and diagnosis.** *Science* 2012, **338:**612-614.

41. Johnson A, Goss A, Beckerman J, Castro A: **Hidden costs: the direct and indirect impact of user fees on access to malaria treatment and primary care in Mali.** *Soc Sci Med* 2012, **75:**1786-1792.

42. Korenromp EL: **Lives saved from malaria prevention in Africa--evidence to sustain cost-effective gains.** *Malar J* 2012, **11:**94.

43. Svihrova V, Szilagyiova M, Novakova E, Svihra J, Hudeckova H: **Costs analysis of the treatment of imported malaria.** *Malar J* 2012, **11:**1.

44. Ezeoke OP, Onwujekwe OE, Uzochukwu BS: **Towards universal coverage: examining costs of illness, payment, and coping strategies to different population groups in southeast Nigeria.** *Am J Trop Med Hyg* 2012, **86:**52-57.

45. Worrall E, Fillinger U: **Large-scale use of mosquito larval source management for malaria control in Africa: a cost analysis.** *Malar J* 2011, **10:**338.

46. White MT, Conteh L, Cibulskis R, Ghani AC: **Costs and cost-effectiveness of malaria control interventions--a systematic review.** *Malar J* 2011, **10:**337.

47. O'Connell KA, Gatakaa H, Poyer S, Njogu J, Evance I, Munroe E, Solomon T, Goodman C, Hanson K, Zinsou C, et al: **Got ACTs? Availability, price, market share and provider knowledge of anti-malarial medicines in public and private sector outlets in six malaria-endemic countries.** *Malar J* 2011, **10:**326.

48. Drake TL, Okello G, Njagi K, Halliday KE, Jukes M, Mangham L, Brooker S: **Cost analysis of school-based intermittent screening and treatment of malaria in Kenya.** *Malar J* 2011, **10:**273.

49. Bisoffi Z, Sirima SB, Meheus F, Lodesani C, Gobbi F, Angheben A, Tinto H, Neya B, Van den Ende K, Romeo A, Van den Ende J: **Strict adherence to malaria rapid test results might lead to a neglect of other dangerous diseases: a cost benefit analysis from Burkina Faso.** *Malar J* 2011, **10:**226.

50. Chanda P, Hamainza B, Moonga HB, Chalwe V, Banda P, Pagnoni F: **Relative costs and effectiveness of treating uncomplicated malaria in two rural districts in Zambia: implications for nationwide scale-up of home-based management.** *Malar J* 2011, **10:**159.

51. malEra Consultative Group on Monitoring: **A research agenda for malaria eradication: monitoring, evaluation, and surveillance.** *PLoS Med* 2011, **8:**e1000400.

52. malERA Consultative Group on Integration Strategies: **A research agenda for malaria eradication: cross-cutting issues for eradication.** *PLoS Med* 2011, **8:**e1000404.

53. Tatarsky A, Aboobakar S, Cohen JM, Gopee N, Bheecarry A, Moonasar D, Phillips AA, Kahn JG, Moonen B, Smith DL, Sabot O: **Preventing the reintroduction of malaria in Mauritius: a programmatic and financial assessment.** *PLoS One* 2011, **6:**e23832.

54. Buchanan J, Mihaylova B, Gray A, White N: **Cost-effectiveness of pre-referral antimalarial, antibacterial, and combined rectal formulations for severe febrile illness.** *PLoS One* 2010, **5:**e14446.

55. Sabot O, Cohen JM, Hsiang MS, Kahn JG, Basu S, Tang L, Zheng B, Gao Q, Zou L, Tatarsky A, et al: **Costs and financial feasibility of malaria elimination.** *The Lancet* 2010, **376:**1604-1615.

56. Oliveira MR, de Castro Gomes A, Toscano CM: **Cost effectiveness of OptiMal(R) rapid diagnostic test for malaria in remote areas of the Amazon Region, Brazil.** *Malar J* 2010, **9:**277.

57. Chizema-Kawesha E, Miller JM, Steketee RW, Mukonka VM, Mukuka C, Mohamed AD, Miti SK, Campbell CC: **Scaling up malaria control in Zambia: progress and impact 2005-2008.** *Am J Trop Med Hyg* 2010, **83:**480-488.

58. Onwujekwe O, Dike N, Uzochukwu B, Ezeoke O: **Informal payments for healthcare: differences in expenditures from consumers and providers perspectives for treatment of malaria in Nigeria.** *Health Policy* 2010, **96:**72-79.

59. Ogutu B, Tiono AB, Makanga M, Premji Z, Gbadoe AD, Ubben D, Marrast AC, Gaye O: **Treatment of asymptomatic carriers with artemether-lumefantrine: an opportunity to reduce the burden of malaria?** *Malar J* 2010, **9:**30.

60. Mosha JF, Conteh L, Tediosi F, Gesase S, Bruce J, Chandramohan D, Gosling R: **Cost implications of improving malaria diagnosis: findings from north-eastern Tanzania.** *PLoS One* 2010, **5:**e8707.

61. Mustafa HS, Malik EM, Tuok HT, Mohamed AA, Julla AI, Bassili A: **Malaria preventive measures, health care seeking behaviour and malaria burden in different epidemiological settings in Sudan.** *Trop Med Int Health* 2009, **14:**1488-1495.

62. Patil AP, Okiro EA, Gething PW, Guerra CA, Sharma SK, Snow RW, Hay SI: **Defining the relationship between Plasmodium falciparum parasite rate and clinical disease: statistical models for disease burden estimation.** *Malar J* 2009, **8:**186.

63. Svihrova V, Szilagyiova M, Krkoska D, Simekova K, Hudeckova H, Avdicova M: **Analysis of the direct and indirect costs of treatment of imported malaria in the Slovak Republic.** *Revista da Sociedade Brasileira de Medicina Tropical* 2009, **42:**377-380.

64. McElroy B, Wiseman V, Matovu F, Mwengee W: **Malaria prevention in north-eastern Tanzania: patterns of expenditure and determinants of demand at the household level.** *Malar J* 2009, **8:**95.

65. Yukich JO, Zerom M, Ghebremeskel T, Tediosi F, Lengeler C: **Costs and cost-effectiveness of vector control in Eritrea using insecticide-treated bed nets.** *Malar J* 2009, **8:**51.

66. Diamond-Smith N, Singh N, Gupta RK, Dash A, Thimasarn K, Campbell OM, Chandramohan D: **Estimating the burden of malaria in pregnancy: a case study from rural Madhya Pradesh, India.** *Malar J* 2009, **8:**24.

67. Mueller DH, Abeku TA, Okia M, Rapuoda B, Cox J: **Costs of early detection systems for epidemic malaria in highland areas of Kenya and Uganda.** *Malar J* 2009, **8:**17.

68. Yukich JO, Lengeler C, Tediosi F, Brown N, Mulligan JA, Chavasse D, Stevens W, Justino J, Conteh L, Maharaj R, et al: **Costs and consequences of large-scale vector control for malaria.** *Malar J* 2008, **7:**258.

69. Mills A, Lubell Y, Hanson K: **Malaria eradication: the economic, financial and institutional challenge.** *Malar J* 2008, **7 Suppl 1:**S11.

70. Jain V, Nagpal AC, Joel PK, Shukla M, Singh MP, Gupta RB, Dash AP, Mishra SK, Udhayakumar V, Stiles JK, Singh N: **Burden of cerebral malaria in central India (2004-2007).** *Am J Trop Med Hyg* 2008, **79:**636-642.

71. Hume JC, Barnish G, Mangal T, Armazio L, Streat E, Bates I: **Household cost of malaria overdiagnosis in rural Mozambique.** *Malar J* 2008, **7:**33.

72. Lubell Y, Hopkins H, Whitty CJ, Staedke SG, Mills A: **An interactive model for the assessment of the economic costs and benefits of different rapid diagnostic tests for malaria.** *Malar J* 2008, **7:**21.

73. Lubell Y, Reyburn H, Mbakilwa H, Mwangi R, Chonya S, Whitty CJ, Mills A: **The impact of response to the results of diagnostic tests for malaria: cost-benefit analysis.** *BMJ* 2008, **336:**202-205.

74. Stein C, Kuchenmuller T, Hendrickx S, Pruss-Ustun A, Wolfson L, Engels D, Schlundt J: **The Global Burden of Disease assessments--WHO is responsible?** *PLoS Negl Trop Dis* 2007, **1:**e161.

75. Kumar A, Valecha N, Jain T, Dash AP: **Burden of malaria in India: retrospective and prospective view.** *Am J Trop Med Hyg* 2007, **77:**69-78.

76. Koram KA, Molyneux ME: **When is "malaria" malaria? The different burdens of malaria infection, malaria disease, and malaria-like illnesses.** *Am J Trop Med Hyg* 2007, **77:**1-5.

77. Kiszewski A, Johns B, Schapira A, Delacollette C, Crowell V, Tan-Torres T, Ameneshewa B, Teklehaimanot A, Nafo-Traore F: **Estimated global resources needed to attain international malaria control goals.** *Bull World Health Organ* 2007, **85:**623-630.

78. Onwujekwe O, Uzochukwu B, Ojukwu J, Dike N, Shu E: **Feasibility of a community health worker strategy for providing near and appropriate treatment of malaria in southeast Nigeria: an analysis of activities, costs and outcomes.** *Acta Trop* 2007, **101:**95-105.

79. Worrall E, Morel C, Yeung S, Borghi J, Webster J, Hill J, Wiseman V, Mills A: **The economics of malaria in pregnancy--a review of the evidence and research priorities.** *Lancet Infect Dis* 2007, **7:**156-168.

80. Larson BA, Amin AA, Noor AM, Zurovac D, Snow RW: **The cost of uncomplicated childhood fevers to Kenyan households: implications for reaching international access targets.** *BMC Public Health* 2006, **6:**314.

81. Eliades MJ, Wolkon A, Morgah K, Crawford SB, Dorkenoo A, Sodahlon Y, Hawley WA, Hightower AW, Kuile FO, Terlouw DJ: **Burden of malaria at community level in children less than 5 years of age in Togo.** *Am J Trop Med Hyg* 2006, **75:**622-629.

82. Onwujekwe O, Ojukwu J, Ezumah N, Uzochukwu B, Dike N: **Socio-economic differences in preferences and willingess to pay for different providers of malaria treatment in southeast Nigeria.** *Am J Trop Med Hyg* 2006, **75:**421-429.

83. Goodman CA, Mutemi WM, Baya EK, Willetts A, Marsh V: **The cost-effectiveness of improving malaria home management: shopkeeper training in rural Kenya.** *Health Policy Plan* 2006, **21:**275-288.

84. Mulligan JA, Mandike R, Palmer N, Williams H, Abdulla S, Bloland P, Mills A: **The costs of changing national policy: lessons from malaria treatment policy guidelines in Tanzania.** *Trop Med Int Health* 2006, **11:**452-461.

85. Wiseman V, McElroy B, Conteh L, Stevens W: **Malaria prevention in The Gambia: patterns of expenditure and determinants of demand at the household level.** *Trop Med Int Health* 2006, **11:**419-431.

86. Morel CM, Lauer JA, Evans DB: **Cost effectiveness analysis of strategies to combat malaria in developing countries.** *BMJ* 2005, **331:**1299.

87. Stevens W, Wiseman V, Ortiz J, Chavasse D: **The costs and effects of a nationwide insecticide-treated net programme: the case of Malawi.** *Malar J* 2005, **4:**22.

88. Croft AM, Winfield CR, Horsfall M, Quarrell MA: **Direct health costs of occupationally acquired malaria in a military population in Europe.** *Occup Med (Lond)* 2005, **55:**128-130.

89. Macauley C: **Aggressive active case detection: a malaria control strategy based on the Brazilian model.** *Soc Sci Med* 2005, **60:**563-573.

90. Mujinja PGM, Makwaya CK, Sauerborn R: **Gender and willingness to pay for insecticides treated bed net in a poor rural area in Tanzania.** *East African Medical Journal* 2004, **81**.

91. Shubhakaran, Jakhar R: **Cost effective treatment of acute, uncomplicated Plasmodium falciparum malaria.** *J Assoc Physicians India* 2004, **52:**1009-1010; author reply 1010.

92. Breman JG, Alilio MS, Mills A: **The intolerable burden of malaria: what's new, what's, needed.** *American Journal of Tropical Medicine and Hygiene* 2002.

93. Savigny D, Binka F: **Monitoring future impact on malaria burden in sub-saharan Africa.** *Am J Trop Med Hyg* 2004, **71:**224-231.

94. Hanson K: **Public and private roles in malaria control: the contributions of economic analysis.** *Am J Trop Med Hyg* 2004, **71:**168-173.

95. Agyepong I, Kangeya-Kayonda J: **Proving practical estiamtes of malaria burden for health planners in resource-poor countries.** *Am J Trop Med Hyg* 2004, **17:**162-167.

96. Jones COH, Williams HA: **The social burden of malaria: what are we measuring?** *Am J Trop Med Hyg* 2004, **71:**156-161.

97. Malaney P, Spielman A, Sachs J: **The malaria gap.** *Am J Trop Med Hyg* 2004, **71:**141-146.

98. Worrall E, Rietveld A, Delacollette C: **The burden of malaria epidemics and cost-effectiveness of interventions in epidemic situations in Africa.** *Am J Trop Med Hyg* 2004, **71:**136-140.

99. Laxminarayan R: **Does reducing malaria improve household living standards?** *Tropical Medicine and International Health* 2004, **9:**267-272.

100. Yadav RS, Bhatt RM, Kohli VK, Sharma VP: **The burden of malaria in Ahmedabad city, India: a retrospective analysis of reported cases and deaths.** *Ann Trop Med Parasitol* 2003, **97:**793-802.

101. Gross M: **Gates foots a malaria bill.** *Curr Biol* 2003, **13:**R820-821.

102. Chima RI, Goodman CA, Mills A: **The economic impact of malaria in Africa: a critical review of the evidence.** *Health Policy* 2003, **63:**17-36.

103. Pang LW, Piovesan-Alves F: **Economic advantage of a community-based malaria management program in the Brazilian Amazon.** *Am J Trop Med Hyg* 2001, **65:**883-886.

104. Utzinger J, Tozan Y, Singer BH: **Efficacy and cost-effectiveness of environmental management for malaria control.** *Trop Med Int Health* 2001, **6:**677-687.

105. Hor DK: **Treating malaria may be too expensive.** *Aust Fam Physician* 2001, **30:**527-528.

106. Kilama WL: **The malaria burden and te need for research and capacity strenthening in Africa.** *American Journal of Tropical Medicine and Hygiene* 2001.

107. Mendis K, Sina BJ, Marchesini P, Carter R: **Roll Back Malaria, World Health Organization.** *Am J Trop Med Hyg* 2001, **64:**97-106.

108. Gallup JL, Sachs JD: **The economic burden of malaria.** In *American Journal of Tropical Medicine and Hygiene*American Society of Tropical Medicine and Hygiene; 2001: 85-96.

109. Booth CM, MacLean JD: **Knowledge, treatment-seeking, and socioeconomic impact of malaria on the Essequibo Coast of Guyana.** *McGill Journal of Medicine* 2001, **6:**17-25.

110. Samba E: **The malaria burden and Africa.** *Am J Trop Med Hyg* 2001, **64:**ii.

111. Sachs J, Malaney P: **The economic and social burden of malaria.** *Nature* 2002, **415:**680-685.

112. Anberber S, Tessema T, Yimer A: **Severe malaria in the under-fives--clinical featrues, management and outcome in a district hospital.** *Ethiop Med J* 2003, **41:**301-310.

113. Lopez AD, Mathers CD, Ezzati M, Jamison DT, Murray CJ: **Global and regional burden of disease and risk factors, 2001: systematic analysis of population health data.** *Lancet* 2006, **367:**1747-1757.

114. Jimoh A, Sofola O, Petu A, Okorosobo T: **Quantifying the economic burden of malaria in Nigeria using the willingness to pay approach.** *Cost Eff Resour Alloc* 2007, **5:**6.

115. Legesse Y, Tegegn A, Belachew T, Tushune K: **Households willingness to pay for long-lasting insecticide treated nets in three urban communities of Assosa Zone, western Ethiopia.** *Ethiopian medical journal* 2007, **45:**353-362.

116. Rosenberg M: **Global child health: burden of disease, achievements, and future challenges.** *Curr Probl Pediatr Adolesc Health Care* 2007, **37:**338-362.

117. Teklehaimanot A, McCord GC, Sachs JD: **Scaling up malaria control in Africa: an economic and epidemiological assessment.** *Am J Trop Med Hyg* 2007, **77:**138-144.

118. Hotez PJ, Bottazzi ME, Franco-Paredes C, Ault SK, Periago MR: **The neglected tropical diseases of Latin America and the Caribbean: a review of disease burden and distribution and a roadmap for control and elimination.** *PLoS Negl Trop Dis* 2008, **2:**e300.

119. Bowie C, Mwase T: **Assessing the use of an essential health package in a sector wide approach in Malawi.** *Health Res Policy Syst* 2011, **9:**4.

120. Egbendewe-Mondzozo A, Musumba M, McCarl BA, Wu X: **Climate change and vector-borne diseases: an economic impact analysis of malaria in Africa.** *Int J Environ Res Public Health* 2011, **8:**913-930.

121. Kahn JG, Harris B, Mermin JH, Clasen T, Lugada E, Grabowksy M, Vestergaard Frandsen M, Garg N: **Cost of community integrated prevention campaign for malaria, HIV, and diarrhea in rural Kenya.** *BMC Health Serv Res* 2011, **11:**346.

122. Manzi F, Abdallah G, Khatibu R, Akweongo P: **Monitoring malaria implementation cost - The case of Artemether-Lumefantrine.** *American Journal of Tropical Medicine and Hygiene* 2011, **85:**417.

123. Wetengere K: **Effects of malaria on household poverty in central Tanzania.** *Journal of Malaria Research* 2011, **2:**63-76.

124. Agyei-Baffour P, Asante BO: **Cost drivers of household treatment of presumptive malaria in home-based management of malaria in Ejisu-Juaben Municipality.** *Value in Health* 2012, **15:**A524.

125. Danielle MYB: **The malaria household cost of children under five years old in burkina faso (West Africa).** *American Journal of Tropical Medicine and Hygiene* 2012, **87:**112.

126. Danielle MYB: **Malaria treatment cost in health system: what is the children under five years old malaria provider cost in burkina faso (West Africa)?** *American Journal of Tropical Medicine and Hygiene* 2012, **87:**111.

127. Fadima IYB, Belemsaga D, Kouanda S, Tinto H, Adjagba A: **Malaria prevention measures expenditures in Burkina Faso: How much do they cost to households?** *American Journal of Tropical Medicine and Hygiene* 2012, **87:**120.

128. Nartey AA, Akweongo P, Akpakli J, Awini E, Appiatse AA, Odonkor G, Ajuik M, Aikins M, Gyapong M: **Who pays for malaria treatment in ghana in the era of health insurance policy?** *American Journal of Tropical Medicine and Hygiene* 2012, **87:**172.

129. Ndiaye M, Pitt C, Conteh L, Ba EH, Sy O, Camara PI, Sokhna C, Ndiaye JL, Gomis JF, Cisse B, et al: **Costing a large-scale implementation of seasonal malaria chemoprevention in children delivered through community health workers in senegal.** *American Journal of Tropical Medicine and Hygiene* 2012, **87:**406.

130. Nartey AA, Akweongo P, Awini E, Darlaba M, Tawiah T, Akpakli J, Sarpong D, Adjuik M, Aikins MK, Binka F, Gyapong M: **Household cost of treating fevers in Ghana.** *American Journal of Tropical Medicine and Hygiene* 2013, **89:**25.

131. Njau JD, Briggs M, Chinkhumba J, Bauleni A, Shah M, Chalira A, Moyo D, Dodoli W, Luhanga M, Sande J, et al: **Household costs of inpatient management of malaria in Malawi.** *American Journal of Tropical Medicine and Hygiene* 2013, **89:**345.

132. Purdy M, Robinson M, Wei K, Rublin D: **The economic case for combating malaria.** *Am J Trop Med Hyg* 2013, **89:**819-823.

133. Umeh JC, Ogbanje CE, Ogaba J: **Economic burden of malaria on farm income among rural households in Benue state, Nigeria.** *American Journal of Tropical Medicine and Hygiene* 2013, **89:**64.

134. Yadav SP, Yadav S, Kuma P, Yadav S: **Knowledge, treatment-seeking behaviour and socio-economic impact of malaria in the desert of Rajasthan, India.** *Southern African Journal of Epidemiology and Infection* 2015, **28:**41-47.

135. Amaral LM, Fernandes RA, Takemoto MLS, Padula AC, Vasconcellos JF, Haas LC, Valle P: **Brazilian program for research and development in neglected diseases: Analyses of hospitalization patterns and costs.** *Value in Health* 2014, **17:**A271.

136. Etim S, Ogbeche J: **Household responses to malaria: illness perception, cost implications and treatment-seeking behavior of mothers in Calabar, Nigeria.** *Malaria Journal* 2014, **13**.

137. Goodman C, Cianci F, Cobos D, Tougher S, Hanson K: **Assessing the cost of private sector act subsidies-the financial and economic costs of the affordable medicines facility-malaria (AMFm) in three African countries.** *American Journal of Tropical Medicine and Hygiene* 2014, **91:**482-483.

138. Shepard DS, Mangesho P, Kihomo RM, Kihombo A, Mugasa J, Malongo A, Mnzava R, Tenu F, Rwiyereka AK, Halasa YA, et al: **Household expenditures for malaria treatment from a population-based survey.** *American Journal of Tropical Medicine and Hygiene* 2014, **91:**498-499.

139. Chaparro-Narváez PE, Diaz D, Castañeda-Orjuela C, de la Hoz F: **Burden of Disease and Economic Impact of Malaria In Colombia, 2012.** *Value in Health* 2015, **18:**A234-A234.

140. Ezenduka CC: **Treatment Costs for Uncomplicated Malaria at a Secondary Health Care Facility In Nigeria.** *Value in Health* 2015, **18:**A236-A236.

141. Larson B, Ngoma T, Silumbe K, Rutagwera MR, Hamainza B, Winters A, Miller J, Scott C: **Evaluating the annual costs of implementing case investigation to support malaria elimination in southern province, Zambia: A micro-costing analysis.** *American Journal of Tropical Medicine and Hygiene* 2015, **93:**472.

142. Mbalabu OB, Bobanga TL, Umesumbu SB, Mantshumba JC, Tshibangu EK: **Direct cost of severe malaria management in pediatric hospital of mbujimayi, democratic republic of Congo.** *American Journal of Tropical Medicine and Hygiene* 2015, **93:**104.

143. Niangaly H, Thuilliez J, Balam A, Sissoko MS, Sanz A, Djimdé AA, Doumbo O: **Costs of malaria treatment between public, community and private healthcare providers in subsidized input conditions.** *American Journal of Tropical Medicine and Hygiene* 2015, **93:**296.

144. Oostvogels AJ, De Wit GA, Jahn B, Cassini A, Colzani E, De Waure C, Kretzschmar ME, Siebert U, Muhlberger N, Mangen MJ: **Use of DALYs in economic analyses on interventions for infectious diseases: a systematic review.** *Epidemiol Infect* 2015, **143:**1791-1802.

145. Scott C, Ngoma T, Larson B, Silumbe K, Hamainza B, Yukich J, Miller JM: **Evaluating the costs of implementing a package of interventions and surveillance systems to support malaria elimination in southern province, zambia: A micro-costing analysis.** *American Journal of Tropical Medicine and Hygiene* 2015, **93:**268.

146. Sicuri E: **Costs associated with malaria in pregnancy in the Brazilian Amazon.** *American Journal of Tropical Medicine and Hygiene* 2015, **93:**298.

147. Castellani J, Nsungwa-Sabiiti J, Mihaylova B, Ajayi IO, Siribie M, Afonne C, Balyeku A, Serme L, Sanou AK, Sombie BS, et al: **Impact of Improving Community-Based Access to Malaria Diagnosis and Treatment on Household Costs.** *Clin Infect Dis* 2016, **63:**S256-S263.

148. Glaser EL, Odhiambo JA, Osewe J, Leo B, Olang G, Bayoh MN, Nandakumar AK, Gimnig JE, Shepard DS, Hamel MJ, Perloff JN: **The catastrophic impact of malaria on households in rural western Kenya.** *American Journal of Tropical Medicine and Hygiene* 2016, **95:**511-512.

149. Rahman R, Lesser A, Mboera L, Kramer R: **Cost of microbial larviciding for malaria control in rural Tanzania.** *Trop Med Int Health* 2016, **21:**1468-1475.

150. Bavinger JC, Wise P, Bendavid E: **The relationship between burden of childhood disease and foreign aid for child health.** *BMC Health Serv Res* 2017, **17:**655.

151. Ezenduka CC, Falleiros DR, Godman BB: **Evaluating the Treatment Costs for Uncomplicated Malaria at a Public Healthcare Facility in Nigeria and the Implications.** *Pharmacoecon Open* 2017, **1:**185-194.

152. Lai S, Li Z, Wardrop NA, Sun J, Head MG, Huang Z, Zhou S, Yu J, Zhang Z, Zhou SS, et al: **Malaria in China, 2011-2015: an observational study.** *Bull World Health Organ* 2017, **95:**564-573.

153. Mohamed HM, Goldman AS, Ghosh M: **The economic burden of malaria cases imported from hispaniola to other non-endemic countries in the Western hemisphere (2007-2013).** *American Journal of Tropical Medicine and Hygiene* 2017, **97:**317.

154. Pariti B: **Analysis of treatment costs of malaria in a tertiary care hospital: an indian perspective.** *Value in Health* 2017, **20**.

155. Sudathip P, S. Kitchakorn, D. Kongkasuriyachai, S. Sawang, D. Sintasath and J. Sine **A case for investment in thailand's malaria elimination program: A cost-benefit analysis study.** *American Journal of Tropical Medicine and Hygiene* 2017, **95**.

156. Castellani J, Mihaylova B, Siribie M, Gansane Z, Ouedraogo AZ, Fouque F, Sirima SB, Evers S, Paulus ATG, Gomes M: **Household costs and time to treatment for children with severe febrile illness in rural Burkina Faso: the role of rectal artesunate.** *Malar J* 2018, **17:**380.

157. Devine A, Battle KE, Howes RE, Price RN, Lubell Y: **Global economic costs due to plasmodium vivax malaria treatment and the cost-benefit of radical cure.** *American Journal of Tropical Medicine and Hygiene* 2018, **99:**338.

158. Devine A, Pasaribu AP, Teferi T, Pham HT, Contantia F, Nguyen TN, Ngo VT, Tran H, Hailu A, Price RN, Lubell Y: **Economic costs of plasmodium vivax episodes: A multi-country comparative analysis using primary trial data.** *American Journal of Tropical Medicine and Hygiene* 2018, **99:**427.

159. Swe KT, Rahman MM, Rahman MS, Saito E, Abe SK, Gilmour S, Shibuya K: **Cost and economic burden of illness over 15 years in Nepal: A comparative analysis.** *PLoS One* 2018, **13:**e0194564.

160. Chijioke-Nwauche IN, Kasso T, Maduka O, Awopeju AT, Oboro IL, Nsirimobu PI, Yaguo Ide LE, Ogoro M, Otto G, Nwauche CA: **Economic burden of malaria in pregnant women in rivers State, Nigeria.** *American Journal of Tropical Medicine and Hygiene* 2019, **101:**310.

161. Kitojo C, Gutman JR, Chacky F, Kigadye E, Mkude S, Mandike R, Mohamed A, Reaves EJ, Walker P, Ishengoma DS: **Estimating malaria burden among pregnant women using data from antenatal care centres in Tanzania: a population-based study.** *The Lancet Global Health* 2019, **7:**e1695-e1705.

162. Ngatu NR, Kanbara S, Renzaho A, Wumba R, Mbelambela EP, Muchanga SMJ, Muzembo BA, Leon-Kabamba N, Nattadech C, Suzuki T, et al: **Environmental and sociodemographic factors associated with household malaria burden in the Congo.** *Malar J* 2019, **18:**53.

163. Tun KM, Levin A, Win ZT, Ko BBT, Aung TZ, Htet SM, Lin MA, Price T, Al Mossawi HJ, Oliphant S, et al: **Cost analysis of malaria control and elimination activities in heterogeneous malaria transmission areas of Myanmar.** *American Journal of Tropical Medicine and Hygiene* 2019, **101:**509-510.

164. Alfonso YN, Lynch M, Mensah E, Piccinini D, Bishai D: **Willingness-to-pay for long-lasting insecticide-treated bed nets: a discrete choice experiment with real payment in Ghana.** *Malar J* 2020, **19:**14.

165. Perú. Ministerio de Salud. Proyecto V: **Impacto económico de la malaria en el Perú.** pp. 127-1271999:127-127.

166. Bolivia. Ministerio de Salud y Previsión Social. Dirección General de Control y Prevención de Enfermedades. Programa Nacional de Control de la Malaria. Escudo E, Prosin, Usaid: **Impacto socioeconomico a nivel familiar de la malaria en Bolivia.** pp. 134-1342002:134-134.

167. Akhavan D: **Análise de custo-efetividade do Projeto de Controle da Malária na Bacia Amazônica (PCMAM): relatório final.** pp. 85-852000:85-85.

168. Martín Á, Aguirre R, Fernando L, Zavalaga L, Trelles De Belaunde M: **Relación costo-efectividad del uso de pruebas rápidas para el diagnóstico de la malaria en la Amazonia peruana.** *Rev Panam Salud Publica/Pan Am J Public Health* 2009, **25**.

169. Haakenstad A, Harle AC, Tsakalos G, Micah AE, Tao T, Anjomshoa M, Cohen J, Fullman N, Hay SI, Mestrovic T, et al: **Tracking spending on malaria by source in 106 countries, 2000–16: an economic modelling study.** *The Lancet Infectious Diseases* 2019, **19:**703-716.
