# Supplementary material for: The economic burden of malaria: a systematic review
Source: Malar J. 2022 Oct 5;21:283. doi: 10.1186/s12936-022-04303-6 (PMC9533489; doi:10.1186/s12936-022-04303-6)
Supplement: Supplementary file 6 — Additional file 6. Funding sources of studies included in the analysis. [file 12936_2022_4303_MOESM6_ESM.docx]

# Funding sources of studies included in the analysis. Some studies were funded by more than one source.

| **Funding source** | **n** | **% studies by funding source** | **References** |
| --- | --- | --- | --- |
| World Bank/UNDP/WHO Special Program for Training and Research in Tropical Diseases | 9 | 19.57 | ^[1-9]^ |
| Bill & Melinda Gates Foundation | 7 | 15.22 | ^[10-16]^ |
| Wellcome Trust | 5 | 10.87 | ^[10, 17-20]^ |
| World Health Organization (WHO) | 4 | 8.70 | ^[7, 9, 20, 21]^ |
| Canadian International Development Research Centre (IDRC) | 3 | 6.52 | ^[8, 9, 22]^ |
| PregVax | 2 | 4.35 | ^[13, 16]^ |
| Centers for Disease Control and Prevention (CDC) | 1 | 2.17 | ^[6]^ |
| President’s Malaria Initiative (PMI) | 1 | 2.17 | ^[23]^ |
| Private companies | 1 | 2.17 | ^[24]^ |
| UNITAID | 1 | 2.17 | ^[23]^ |
| National funding | 4 | 8.70 | ^[19, 25-27]^ |
| Other external funding | 12 | 28.26 | ^[6, 8, 9, 16, 17, 27-33]^ |
| No information | 11 | 23.91 | ^[34-44]^ |

1. Gatton ML, Cho Min N: **Costs to the patient for seeking malaria care in Myanmar.** *Acta Trop* 2004, **92:**173-177.

2. Jackson S, Sleigh AC, Liu XL: **Cost of malaria control in China: Henan's consolidation programme from community and government perspectives.** *Bull World Health Organ* 2002, **80:**653-659.

3. Onwujekwe O, Chima R, Okonkwo P: **Economic burden of malaria illness on households versus that of all other illness episodes: a study in five malaria holo-endemic Nigerian communities.** *Health Policy* 2000, **54:**143-159.

4. Attanayake N, Fox-Rushby J, Mills A: **Household costs of 'malaria' morbidity: a study in Matale district, Sri Lanka.** *Tropical Medicine and International Health* 2000, **5:**595-606.

5. Castillo-Riquelme M, McIntyre D, Barnes K: **Household burden of malaria in South Africa and Mozambique: is there a catastrophic impact?** *Trop Med Int Health* 2008, **13:**108-122.

6. Hennessee I, Chinkhumba J, Briggs-Hagen M, Bauleni A, Shah MP, Chalira A, Moyo D, Dodoli W, Luhanga M, Sande J, et al: **Household costs among patients hospitalized with malaria: evidence from a national survey in Malawi, 2012.** *Malar J* 2017, **16:**395.

7. Deressa W, Hailemariam D, Ali A: **Economic costs of epidemic malaria to households in rural Ethiopia.** *Trop Med Int Health* 2007, **12:**1148-1156.

8. Gunda R, Shamu S, Chimbari MJ, Mukaratirwa S: **Economic burden of malaria on rural households in Gwanda district, Zimbabwe.** *Afr J Prim Health Care Fam Med* 2017, **9:**e1-e6.

9. Rashed S, Johnson H, Dongier P, Moreau R, Lee C, Lambert J, Schaefer C: **Economic impact of febrile morbidity and use of permethrin-impregnated bed nets in a malarious area I: study of demographics, morbidity, and household expenditures associated with febrile morbidity in the Republic of Benin.** *Am J Trop Med Hyg* 2000, **62:**173-180.

10. Devine A, Pasaribu AP, Teferi T, Pham HT, Awab GR, Contantia F, Nguyen TN, Ngo VT, Tran TH, Hailu A, et al: **Provider and household costs of Plasmodium vivax malaria episodes: a multicountry comparative analysis of primary trial data.** *Bull World Health Organ* 2019, **97:**828-836.

11. Ewing VL, Lalloo DG, Phiri KS, Roca-Feltrer A, Mangham LJ, SanJoaquin MA: **Seasonal and geographic differences in treatment-seeking and household cost of febrile illness among children in Malawi.** *Malar J* 2011, **10:**32.

12. Tawiah T, Asante KP, Dwommoh RA, Kwarteng A, Gyaase S, Mahama E, Abokyi L, Amenga-Etego S, Hansen K, Akweongo P, Owusu-Agyei S: **Economic costs of fever to households in the middle belt of Ghana.** *Malar J* 2016, **15:**68.

13. Sicuri E, Bardaji A, Sanz S, Alonso S, Fernandes S, Hanson K, Arevalo-Herrera M, Menendez C: **Patients' costs, socio-economic and health system aspects associated with malaria in pregnancy in an endemic area of Colombia.** *PLoS Negl Trop Dis* 2018, **12:**e0006431.

14. Sicuri E, Davy C, Marinelli M, Oa O, Ome M, Siba P, Conteh L, Mueller I: **The economic cost to households of childhood malaria in Papua New Guinea: a focus on intra-country variation.** *Health Policy Plan* 2012, **27:**339-347.

15. Onwujekwe O, Hanson K, Uzochukwu B, Ichoku H, Ike E, Onwughalu B: **Are malaria treatment expenditures catastrophic to different socio-economic and geographic groups and how do they cope with payment? A study in southeast Nigeria.** *Trop Med Int Health* 2010, **15:**18-25.

16. Botto-Menezes C, Bardaji A, Dos Santos Campos G, Fernandes S, Hanson K, Martinez-Espinosa FE, Menendez C, Sicuri E: **Costs Associated with Malaria in Pregnancy in the Brazilian Amazon, a Low Endemic Area Where Plasmodium vivax Predominates.** *PLoS Negl Trop Dis* 2016, **10:**e0004494.

17. Karyana M, Devine A, Kenangalem E, Burdarm L, Poespoprodjo JR, Vemuri R, Anstey NM, Tjitra E, Price RN, Yeung S: **Treatment-seeking behaviour and associated costs for malaria in Papua, Indonesia.** *Malar J* 2016, **15:**536.

18. Somi MF, Butler JR, Vahid F, Njau JD, Kachur SP, Abdulla S: **Economic burden of malaria in rural Tanzania: variations by socioeconomic status and season.** *Trop Med Int Health* 2007, **12:**1139-1147.

19. Chuma JM, Thiede M, Molyneux CS: **Rethinking the economic costs of malaria at the household level: evidence from applying a new analytical framework in rural Kenya.** *Malar J* 2006, **5:**76.

20. Ayieko P, Akumu AO, Griffiths UK, English M: **The economic burden of inpatient paediatric care in Kenya: household and provider costs for treatment of pneumonia, malaria and meningitis.** *Cost Eff Resour Alloc* 2009, **7:**3.

21. Mustafa MH, Babiker MA: **Economic cost of malaria on households during a transmission season in Khartoum State, Sudan.** *East Mediterr Health J* 2007, **13:**1298-1307.

22. Uguru NP, Onwujekwe OE, Uzochukwu BS, Igiliegbe GC, Eze SB: **Inequities in incidence, morbidity and expenditures on prevention and treatment of malaria in southeast Nigeria.** *BMC Int Health Hum Rights* 2009, **9:**21.

23. Alonso S, Chaccour CJ, Elobolobo E, Nacima A, Candrinho B, Saifodine A, Saute F, Robertson M, Zulliger R: **The economic burden of malaria on households and the health system in a high transmission district of Mozambique.** *Malar J* 2019, **18:**360.

24. Sicuri E, Vieta A, Lindner L, Constenla D, Sauboin C: **The economic costs of malaria in children in three sub-Saharan countries: Ghana, Tanzania and Kenya.** *Malar J* 2013, **12:**307.

25. Tang S, Feng D, Wang R, Ghose B, Hu T, Ji L, Wu T, Fu H, Huang Y, Feng Z: **Economic burden of malaria inpatients during National Malaria Elimination Programme: estimation of hospitalization cost and its inter-province variation.** *Malar J* 2017, **16:**291.

26. Xia S, Ma JX, Wang DQ, Li SZ, Rollinson D, Zhou SS, Zhou XN: **Economic cost analysis of malaria case management at the household level during the malaria elimination phase in The People's Republic of China.** *Infect Dis Poverty* 2016, **5:**50.

27. Moreno-Gutierrez D, Rosas-Aguirre A, Llanos-Cuentas A, Bilcke J, Barboza JL, Hayette MP, Contreras-Mancilla J, Aguirre K, Gamboa D, Rodriguez H, et al: **Economic costs analysis of uncomplicated malaria case management in the Peruvian Amazon.** *Malar J* 2020, **19:**161.

28. Dalaba MA, Welaga P, Oduro A, Danchaka LL, Matsubara C: **Cost of malaria treatment and health seeking behaviour of children under-five years in the Upper West Region of Ghana.** *PLoS One* 2018, **13:**e0195533.

29. Hailu A, Lindtjorn B, Deressa W, Gari T, Loha E, Robberstad B: **Economic burden of malaria and predictors of cost variability to rural households in south-central Ethiopia.** *PLoS One* 2017, **12:**e0185315.

30. Morel CM, Thang ND, Xa NX, Hung le X, Thuan le K, Van Ky P, Erhart A, Mills AJ, D'Alessandro U: **The economic burden of malaria on the household in south-central Vietnam.** *Malar J* 2008, **7:**166.

31. Jowett M, Miller NJ: **The financial burden of malaria in Tanzania: implications for future government policy.** *Int J Health Plann Manage* 2005, **20:**67-84.

32. Dalaba MA, Akweongo P, Aborigo RA, Saronga HP, Williams J, Aninanya GA, Sauerborn R, Loukanova S: **Cost to households in treating maternal complications in northern Ghana: a cross sectional study.** *BMC Health Serv Res* 2015, **15:**34.

33. Onwujekwe O, Uguru N, Etiaba E, Chikezie I, Uzochukwu B, Adjagba A: **The economic burden of malaria on households and the health system in Enugu State southeast Nigeria.** *PLoS One* 2013, **8:**e78362.

34. Onyia VU, Ughasoro MD, Onwujekwe OE: **The economic burden of malaria in pregnancy: a cross-sectional study.** *J Matern Fetal Neonatal Med* 2020, **33:**92-95.

35. Singh MP, Saha KB, Chand SK, Sabin LL: **The economic cost of malaria at the household level in high and low transmission areas of central India.** *Acta Trop* 2019, **190:**344-349.

36. Beogo I, Huang N, Drabo MK, Ye Y: **Malaria related care-seeking-behaviour and expenditures in urban settings: A household survey in Ouagadougou, Burkina Faso.** *Acta Trop* 2016, **160:**78-85.

37. Ilunga-Ilunga F, Leveque A, Okenge Ngongo L, Tshimungu Kandolo F, Dramaix M: **Costs of treatment of children affected by severe malaria in reference hospitals of Kinshasa, Democratic Republic of Congo.** *J Infect Dev Ctries* 2014, **8:**1574-1583.

38. Ray G, Okogun A, Amadi, A N: **Epidemiology, therapeutic agents and cost of management of paediatric malaria in a Nigerian tertiary hospital.** *J Vect Borne Dis* 2005, **42:**87-94.

39. Mugisha F, Kouyate B, Gbangou A, Sauerborn R: **Examining out-of-pocket expenditure on health care in Nouna, Burkina Faso: implications for health policy.** *Trop Med Int Health* 2002, **7:**187-196.

40. Abdel-Hameed AA, Abdalla HM, Alnaury AH: **Household expenditure on malaria case management in Wad-Medani, Sudan.** *Afr J Med Med Sci* 2001, **30 Suppl:**35-38.

41. Yusuf WA, Yusuf SA, Oladunni OS: **Financial Burden of Malaria Treatment by Households in Northern Nigeria.** *Afr J Biomed Res* 2019, **22:**11-18.

42. Kodhiambo MO, Oyugi JO, Amugune BK: **Modelling the household cost of paediatric malaria treatment in a rural county in Kenya: do non-user fee payments matter? A partial cost of illness analysis.** *BMJ Open* 2020, **10:**e033192.

43. Tefera DR, Sinkie SO, Daka DW: **Economic Burden of Malaria and Associated Factors Among Rural Households in Chewaka District, Western Ethiopia.** *Clinicoecon Outcomes Res* 2020, **12:**141-152.

44. Fernández García A, Collazo Herrera M, Mendes NP, Hossi JP: **Costos directos sanitarios del paludismo en el Hospital Militar Regional de Uíge, Angola Direct health costs of malaria in the Regional Military Hospital of Uíge, Angola.** *Medisur* 2018, **16:**6-09.
