# Supplementary material for: The economic burden of malaria: a systematic review
Source: Malar J. 2022 Oct 5;21:283. doi: 10.1186/s12936-022-04303-6 (PMC9533489; doi:10.1186/s12936-022-04303-6)
Supplement: Supplementary file 7 — Additional file 7. Distribution of selected papers according to the type of stratification. [file 12936_2022_4303_MOESM7_ESM.docx]

# Distribution of selected papers according to the type of stratification

| **Type of Stratification** | **No (n)** | **%** | **Yes (n)** | **%** |
| --- | --- | --- | --- | --- |
| Age group | 24 | 80.0 | 6 | 20.0 |
| Socioeconomic | 26 | 86.7 | 4 | 13.3 |
| Type of service | 23 | 76.7 | 7 | 23.3 |
| Distance to hospital | 29 | 96.7 | 1 | 3.3 |
| Parasite type | 24 | 80.0 | 6 | 20.0 |
| Inpatient/Outpatient Care | 18 | 60.0 | 12 | 40.0 |
| Disease Severity | 29 | 96.7 | 1 | 3.3 |
| Raining period | 26 | 86.7 | 4 | 13.3 |
| Endemic Area | 22 | 73.3 | 8 | 26.7 |
| Region/Country | 19 | 63.3 | 11 | 36.7 |
| Urban/Rural | 27 | 90.0 | 3 | 10.0 |

Note: 15 papers did not stratify the cost results.
