# Supplementary material for: The economic burden of malaria: a systematic review
Source: Malar J. 2022 Oct 5;21:283. doi: 10.1186/s12936-022-04303-6 (PMC9533489; doi:10.1186/s12936-022-04303-6)
Supplement: Supplementary file 8 — Additional file 8. Quality assessment of the selected papers. [file 12936_2022_4303_MOESM8_ESM.docx]

# Quality assessment of the selected papers

| **Items Assessed** | **Number of articles** | | | |
| --- | --- | --- | --- | --- |
|  | **Fully meet the item** | **Partially meet the item** | **Did not meet the item** | **Not applicable** |
| Objective clearly stated and properly answered | 40 | 0 | 5 | 0 |
| Target population clearly described | 38 | 2 | 4 | 1 |
| Study perspective stated | 41 | 1 | 3 | 0 |
| Method for costs estimation clearly described | 45 | 0 | 1 | 0 |
| Cost components in line with the study perspective | 44 | 0 | 1 | 0 |
| Cost components clearly described | 30 | 2 | 13 | 0 |
| Presence of Information on the currency and the period in which the costs were collected | 36 | 5 | 4 | 0 |
| Adjustment for inflation | 5 | 4 | 2 | 34 |
| Cost components results presented in a disaggregated way | 33 | 7 | 5 | 0 |
